# SEPROGADIC – serum protein-based gastric cancer prediction model for prognosis and selection of proper adjuvant therapy

**DOI:** 10.1038/s41598-018-34858-x

**Published:** 2018-11-15

**Authors:** Hee-Sung Ahn, Tae Sung Sohn, Mi Jeong Kim, Byoung Kyu Cho, Su Mi Kim, Seung Tae Kim, Eugene C. Yi, Cheolju Lee

**Affiliations:** 1 Center for Theragnosis, Korea Institute of Science and Technology, 5 Hwarangro-14-gil, Seongbuk-gu, Seoul 02792 Republic of Korea; 2 Division of Bio-Medical Science & Technology, KIST School, Korea University of Science and Technology, 5 Hwarangro-14-gil, Seongbuk-gu Seoul, 02792 Republic of Korea; 3 Department of Surgery, Samsung Medical Center, Sungkyunkwan University School of Medicine, 81 Irwon-ro, Gangnam-gu, Seoul 06351 Republic of Korea; 4 Department of Molecular Medicine and Biopharmaceutical Sciences, School of Convergence Science and Technology and College of Medicine, Seoul National University, 103 Daehak-ro, Jongno-gu, Seoul 03080 Republic of Korea; 5 Department of Medicine, Samsung Medical Center, Sungkyunkwan University School of Medicine, 81 Irwon-ro, Gangnam-gu, Seoul 06351 Republic of Korea; 6 KHU-KIST Department of Converging Science and Technology, Kyung Hee University, 26 Kyunghee-daero, Dongdaemun-gu, Seoul 02447 Republic of Korea

## Abstract

Gastric cancer (GC) patients usually receive surgical treatment. Postoperative therapeutic options such as anticancer adjuvant therapies (AT) based on prognostic prediction models would provide patient-specific treatment to decrease postsurgical morbidity and mortality rates. Relevant prognostic factors in resected GC patient’s serum may improve therapeutic measures in a non-invasive manner. In order to develop a GC prognostic model, we designed a retrospective study. In this study, serum samples were collected from 227 patients at a 4-week recovery period after D2 lymph node dissection, and 103 cancer-related serum proteins were analyzed by multiple reaction monitoring mass spectrometry. Using the quantitative values of the serum proteins, we developed SEPROGADIC (SErum PROtein-based GAstric cancer preDICtor) prognostic model consisting of 6 to 14 serum proteins depending on detailed purposes of the model, prognosis prediction and proper AT selection. SEPROGADIC could clearly classify patients with good or bad prognosis at each TNM stage (1b, 2, 3 and 4) and identify a patient subgroup who would benefit from CCRT (combined chemoradiation therapy) rather than CTX (chemotherapy), or vice versa. Our study demonstrated that serum proteins could serve as prognostic factors along with clinical stage information in patients with resected gastric cancer, thus allowing patient-tailored postsurgical treatment.

## Introduction

The death toll of gastric cancer (GC) was about 819,000 worldwide in 2015, ranking the third in mortality. It was 13,000 in South Korea^1^. Surgical intervention with D1 or D2 lymphadenectomy has been the standard of care option for GC patients. Additionally, adjuvant therapies (AT) are accepted internationally to improve treatment outcome of cancer^2^. Different options for lymphadenectomy and AT have been tested in various clinical trials, including SWOG/INT-0116^3^, MAGIC^4^, NCC^5^, and ARTIST (Adjuvant chemoRadioTherapy In Stomach Tumors)^6^. Despite toxicity issue, D2 lymph node dissection has been found to be more effective than D1 dissection in the east^7^ and west^8^. New drugs and AT methods have been continuously developed to increase patients’ survival time^2^.

Besides treatments, various diagnostic techniques have also been developed to guide efficient remedy for patients^9,10^. A blood test is a basic medical action for cancer patients. Blood biomarkers may play an important role in monitoring treatment progress and condition of patients. Despite the availability of blood biomarkers, their clinical interventional roles have been insufficient in current medical situation^11^. To connect medical needs with unknown biomarkers, we took advantage of mass spectrometry (MS)-based approaches to discover multi-marker signatures of large cohorts of patients with surgical treatment and follow-up AT response^12^.

**Figure 1:**
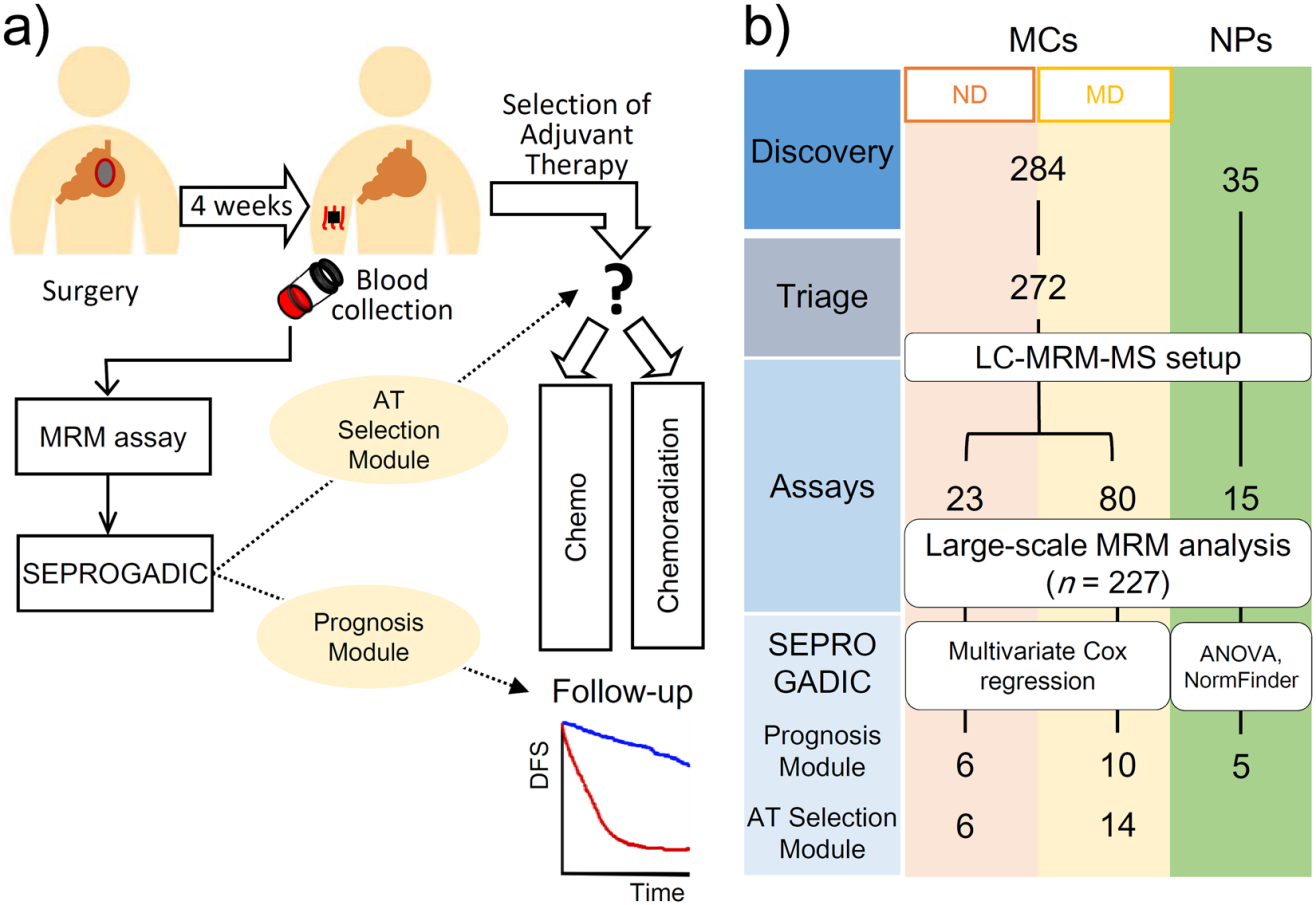
Study workflow and design. (**a**) Standard care for gastric cancer patients. MRM assay is performed on serum collected after surgery and used to build SEPROGADIC prognosis models. (**b**) Multistage MRM-MS workflow for discovery, triage, assays, and prognosis module-building. The 284 marker candidates were obtained by combining data from literature search and MS/MS profiling of GC plasma. MC: Marker Candidate; NP: Normalization Protein; ND: Non-Depletion; MD: MARS14 affinity column Depletion of high abundant proteins.

In this study, we analyzed serum proteome of 227 gastric patients previously involved in the ARTIST trial. We quantified a total of 93 serum biomarker candidates by multiple reaction monitoring (MRM)-MS, composed multi-marker panels using quantification results, and built a disease prediction model SEPROGADIC (SErum PROtein-based GAstric cancer preDICtor) to help predict the prognosis and suggest suitable AT for patients postoperatively. SEPROGADIC can stratify high or low risk groups in combination with clinical stage values and evaluate population of patients who could benefit from CTX or CCRT as adjuvant modalities (Fig. 1).

## Results

### Sets of GC serum biomarker candidates

To identify GC biomarker candidates for LC-MRM-MS validation process, we conducted literature data mining to create potential list of GC biomarkers, including proteins that were expressed differentially between GC tissues and matched normal tissues^13^, differentially secreted proteins between GC cells and normal cells^14^, serum proteins expressing in different levels between healthy controls and patients with locally advanced or metastatic GC^15^, and proteins that corresponded to genes expressed differentially between GC tissues and normal tissues^16^. In the meantime, we took small aliquots from serum samples used in this study, combined them at the same ratio, and profiled constituting proteins by LC-MS/MS. For deep-down profiling, we carried out prefractionation by basic reversed phase liquid chromatography (bRPLC) and two LC-MS/MS runs for each prefractionated sample as described in Methods. A total of 1,013 proteins were identified. Comparing these proteins to proteins collected from published data as stated above, 284 proteins overlapping between the two datasets were chosen as initial serum biomarker candidates.

### Serum sample preparations and development of LC-MRM-MS assays

The complexity of serum proteins with a wide dynamic range hampers simultaneous detection of high and low-abundant proteins by mass spectrometry^17^. To improve dynamic detection of serum proteome, we adopted two independent sample preparations, MARS14 affinity column depletion of 14 high abundant proteins (MD) or non-depletion (ND), followed by in-solution digestion. We also determined MRM surrogate peptides of protein candidates as follows. Except for eight proteins for which no unique peptide could be found, we selected MRM transitions for unique peptides of the remaining 272 proteins by using an *in-house* spectrum library constructed through multiple plasma proteome analyses or public spectrum library such as SRMAtlas^18^ and PeptideAtlas^19^. For these 272 proteins, 589 unique peptides with up to four peptides per protein were synthesized and used to optimize MRM transition parameters (Supplementary Table S1).

MRM signal intensities of most protein candidates were increased in MD serum samples^11^. However, some proteins showed decreased intensities presumably due to interactions with MARS14-depleted proteins. Since not all peptides could be detected by a single 60-min LC-MRM-MS run, we classified proteins into one of two panels based on their signal intensities. Proteins detected in only MD sample preparation were classified as MD panels while proteins detected in both sample preparations or proteins showing decreased intensities in MD sample preparations were classified as ND panels. Proteins monitored in MD but not in ND included MARS14 target proteins and proteins whose concentrations were perturbed by MARS14 depletion. Finally, the ND panel contained 35 peptides corresponding to 23 proteins while the MD panel contained 130 peptides corresponding to 80 proteins.

We chose internal standards from endogenous proteins for data normalization of MRM assay results of target proteins. Internal standard proteins had to fulfil several conditions; (1) such proteins should not be quantitatively vulnerable in GC pathological conditions or affected by MARS14 depletion process; (2) their concentration ranges should not be more than several tens of μg/mL to be detected in both MD and ND samples. Based on these criteria, we filtered out 35 proteins in Plasma Proteome Database (PPD)^20^ as candidates of normalizing proteins. In a preliminary LC-MRM-MS in which representative proteotypic peptides (one per each protein) were monitored, fifteen candidates showed peak intensities greater than 20,000 in both MD and ND samples, which met our criteria of detectability. These fifteen peptides were included in the main LC-MRM-MS analysis.

### Characteristics of samples used in the study design

In this retrospective cohort study, we analyzed serum proteomes of 227 gastric patients previously involved in the ARTIST trial. Their demographic and clinical variables are summarized in Table 1. Prior to MRM-MS, we performed Kaplan-Meier (KM) survival analysis in terms of age (younger vs. older than 50 years), gender, clinical stage, type of AT, tumor location, and histological classification (Supplementary Table S2). Among them, both clinical stage and type of AT were statistically significant (P < 0.05) with disease-free survival (DFS). Hazard ratios (HRs) for cancer recurrence were increased according to clinical stage value (14.41; 1b vs. 4). Patients treated by CTX showed marginally poorer prognosis than patients treated by CCRT (P = 0.042; HR = 1.22). Other clinical variables were not significantly relevant to cancer recurrence.

**Table 1:** Demographic and clinical variables of 227 GC patients.

Clinical variables	Patient No (%)
**Sex (Number)**	
Male	70 (30.84)
Female	157 (69.16)
**Age (years)**	
Median	52
Interquartile range	45–59
**Disease-free survival (months)**	
Median	69.4
Interquartile range	63.88–81.5
**Overall survival (months)**	
Median	71.6
Interquartile range	65.13–82.07
**TNM staging (Number)**	
Ib	58 (25.55)
II	88 (38.77)
III	61 (26.87)
IV	20 (8.81)
**Lymph node dissection (Number)**	
D2	227 (100)
**Lauren classification (Number)**	
Intestinal	153 (67.40)
Diffuse	70 (30.84)
Unknown	4 (1.76)
**Histological grade**	
Well to moderate adenocarcinoma	55 (24.23)
Poor to undifferentiated adenocarcinoma	81 (35.68)
Lymphoepitheliomatous carcinoma	3 (1.32)
Mixed adeno-neuroendocrine carcinoma	1 (0.44)
Mucinous adenocarcinoma	11 (4.85)
Papillary adenocarcinoma	10 (4.41)
Signet-ring cell carcinoma	66 (29.07)
**Tumor primary site (Number)**	
Antrum	93 (40.97)
Angle	1 (0.44)
Body	119 (52.42)
Cardia	10 (4.41)
Fundus	1 (0.44)
Pylorus	3 (1.32)
Regional lymph nodes classification*	
N0	34 (40.97)
N1	190 (40.97)
N2	1 (0.44)
Unknown	2 (0.88)
**ECOG performance status**	
0	111 (48.90)
1	114 (50.22)
2	0 (0.00)
3	0 (0.00)
4	0 (0.00)
5	0 (0.00)
Unknown	2 (0.88)
**Post-operation treatment (Number)**	
Chemotherapy	107 (47.14)
Combined chemo-radiation therapy	120 (52.86)

^*^American Joint Committee on Cancer Staging System, 6th edition (2002).

### Large-scale MRM analysis of 227 GC sera

The optimized MRM assay was applied to the 227 GC patient sera. To minimize possible systematic errors that might be introduced by sequential sample analysis, we designed multiple batch-run processes for sample analysis (An analytical batch contained 21–24 clinical samples plus one quality control sample (a standard sample made by mixing all sera at the same volume ratio). Clinical variables such as AT, stage, age, gender, tumor location, and histology were not significantly different between batches according to sample configuration (P > 0.05; ANOVA for age and Chi-squared test for other variables). In each batch run, two samples were randomly selected and measured three times. Median coefficient of variance (CV) of raw peak areas for all peptides in triplicated twenty samples (10 batches × 2 samples per batch) was 5.26% for ND panel (35 + 15 peptides of 20 samples) and 7.01% for MD panel (130 + 15 peptides for 20 samples). If a peptide showed CV value greater than 25% in more than five samples, the peptide was excluded from further data analysis (Supplementary Table S3).

**Figure 2:**
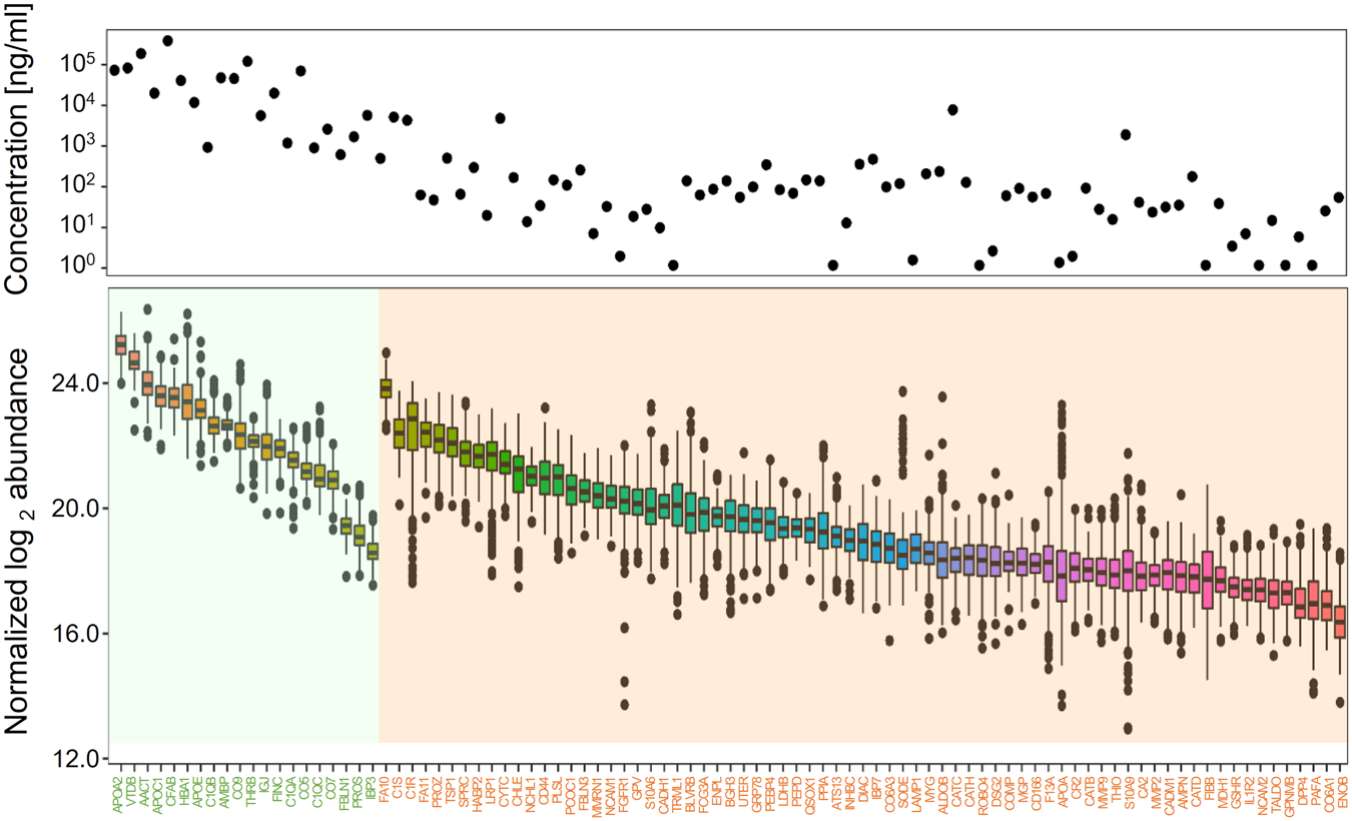
Serum protein abundances in Plasma Proteome Database (top) and normalized protein amount measured by MRM-MS (bottom). The first 20 proteins are ND panel proteins and the rest 73 proteins are MD panel proteins. Data are presented with box plots.

Among the monitored 15 normalizing protein (NP) candidates, we selected the best five NPs by using NormFinder program^21^. We first performed ANOVA test for all 15 peptides between three patient groups consisting of patients recurred within 6 years (*n* = 44), patients censored before 6 years (*n* = 80), and patients without recurrence until 6 years (*n* = 103). Of peptides with ANOVA P-value of 0.05 or greater, five peptides (representative peptides of AZGP1, CLU, ITIH1, KNG1, and SERPINF2) with the best stability value (i.e., least perturbed) in NormFinder were selected. We generated normalization scaling factors (NSFs) of LC-MRM-MS runs using these five NPs. Resultant NSFs were further fine-tuned by using the result of ten-plex replicated QC sample to correct deviation of ionization efficiency of each specific peptide (see details in Methods; Supplementary Table S4). After normalization, we observed reduction in instrumental response variation of QC samples over time. Although we monitored several peptides per protein during LC-MRM-MS, we quantified and processed one representative peptide for each protein (20 in ND panel and 73 in MD panel) with the following order of selection criteria: (1) smallest interference from neighboring signals, (2) lowest CVs in triplicated 20 samples, and (3) highest peak areas (Supplementary Table S4). The normalized peak area of the 93 proteins was highly correlated with the known serum concentration in PPD (*r* = 0.69, Pearson correlation coefficient; Fig. 2).

### Univariate survival analysis

In order to determine whether the amount of single serum protein correlated with the recurrence of gastric cancer, we performed univariate survival analysis for all detected proteins at five time points, from 2 to 6 years of postsurgical patient treatment (PPT) by using survivalROC^22,23^ (Supplementary Table S5). The same statistical analysis was also performed on the TNM stage value, a standardized benchmark for classifying GC patients into low- and high- risk groups to determine an appropriate treatment (https://cancerstaging.org). Within 2 years of PPT, complement component 5 was a better prognostic marker than stage information and four proteins (PROS, CATD, CO7 and CO6A1 (>0.708)) showed similar performance to stage information. At 3 years or after PPT, stage information was the best. None of these 227 patients had a recurrence after 6 years of PPT. Thus, survival AUC value did not change thereafter. Based on analysis at 6 years of PPT, we stratified good and bad prognostic groups with a cutoff abundance value of individual 93 proteins. Of these, 26 proteins were statistically significant in classifying patient subtypes as low or high risk group (log-rank test: P < 0.05). This demonstrates their usefulness as prognostic biomarkers of gastric cancer. However, no protein surpasses TNM stage information in predicting recurrence within six years.

### Overview of the study design - SEPROGADIC

SEPROGADIC was designed to address two major clinical problems faced by patients after lymphadenectomy. The first is a question about better prognosis prediction than a single TNM stage information to monitor high-risk patients and the second is about choosing a more efficient AT for patients who underwent surgery (Fig. 1a). Accordingly, we have built appropriate modules, prognosis module and AT selection module, based on serum protein quantities through feature selection process (Fig. 1b).

### SEPROGADIC – prognosis module

**Figure 3:**
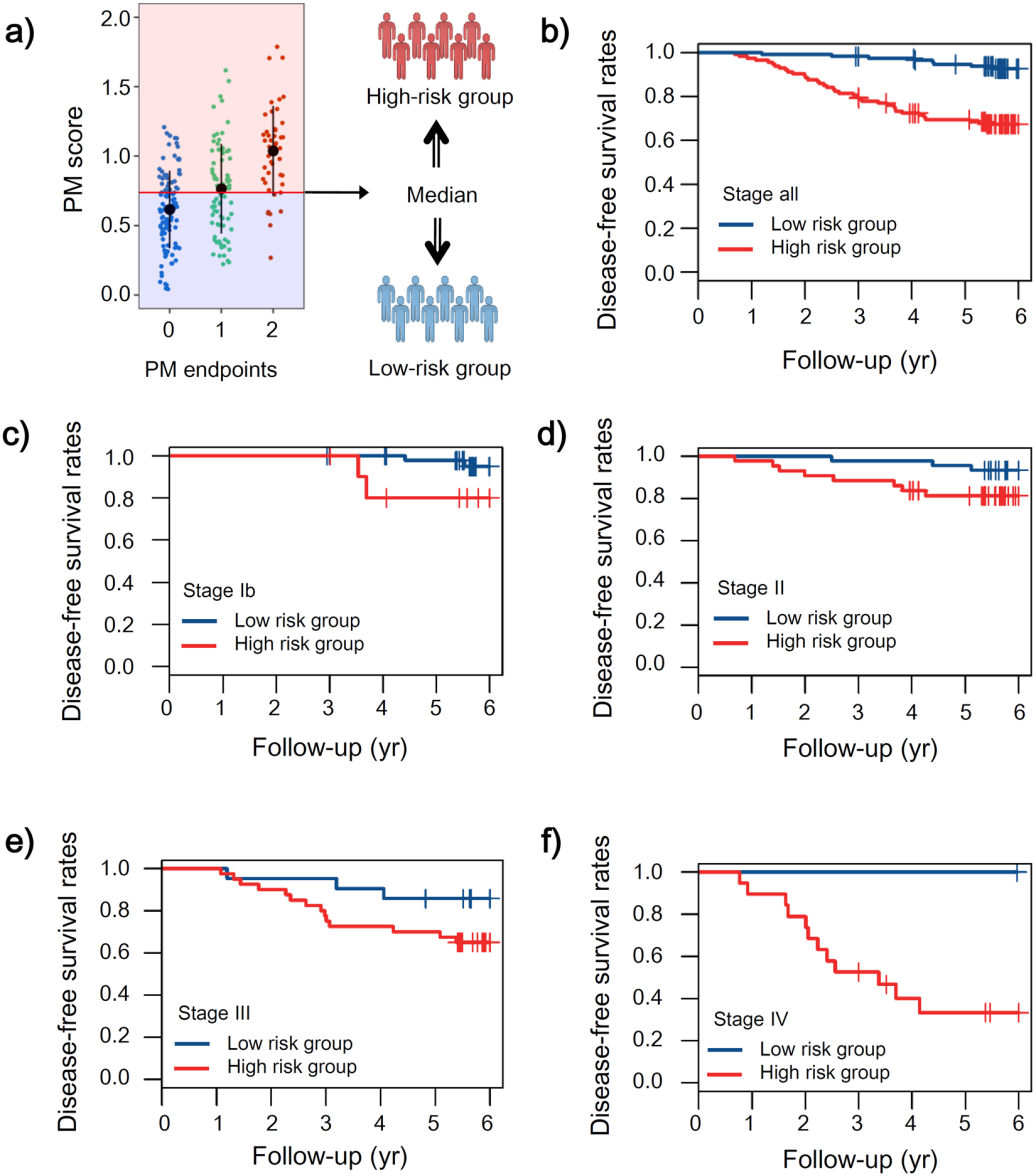
Kaplan-Meier plots of MD panel in SEPROGADIC prognostic module. (**a**) Classification of all the patients into two risk groups by the median of prognostic module (PM) scores. (**b**) All patients: low risk group (n = 114; observed: 7), high risk group (n = 113; observed: 37), P = 1e-07. (**c**) Patients at stage 1b: low risk group (n = 47; observed: 2), high risk group (n = 11; observed: 2), P = 0.063. (**d**) Patients at stage 2: low risk group (n = 44; observed: 2), high risk group (n = 44; observed: 9, P = 0.021. (**e**) Patients at stage 3: low risk group (n = 22; observed: 3), high risk group (n = 39; observed: 14), P = 0.069. (**f**) Patients at stage 4: low risk group (n = 1; observed: 0), high risk group (n = 19; observed: 12), P = 0.308.

TNM stage has been a major classifier for low- and high- risk groups determined by recurrence or survival time. Unfortunately, this grouping alone, usually four groups, can hardly reflect individual patient’s or subtypes properties within the group. We developed SEPROGADIC-prognosis module to improve patient subtyping by combining TNM with protein biomarkers. For this, we first defined prognostic endpoints based on 6-year DFS as described in Methods and performed Cochran-Mantel-Haenszel (CMH) test for possible association of clinical stage with AT, gender, tumor location, ECOG performance status, or age (younger vs older than 50)^24^. Except for histology (P < 0.05), other factors were not significantly associated with clinical stage. To control the confounding effect of clinical stage, we built our module by using multivariate analysis incorporating the clinical stage variable as well as protein features and decided to stratify the data later according to clinical stage. To select representing proteins to relevant features, we performed Cox proportional-hazards regression analysis on 6-year DFS data with backward elimination steps. The analysis was iterated 500 times with 8-fold cross-validation and proteins selected more than four times out of eight in each iteration were counted. In the MD panel, ten proteins (CATC, CATD, CD166, FA10, FA11, IBP7, NCAM1, PLSL, ROBO4 and TRML1) and stage were selected. Their AUC value was 0.792 (95% CI: 0.773–0.802, BCa 2001 bootstrapping analysis) in 6-year Cox regression analysis (Supplementary Fig. S1A and Table 2). In the ND panel, six proteins (C1QA, CO5, CO7, CO9, FBLN1, and THRB) plus stage were selected. Their AUC value was 0.814 (95% CI: 0.800–0.830; Supplementary Fig. S1B and Table 2). These prognostic modules were more significant than the stage-only model (AUC: 0.724) (Likelihood ratio test: P < 0.001), increasing the AUC value by 0.07~0.09. The classifier scores generated by the prognosis module (PM score; Supplementary Table S6) showed the high sensitivities (82%: MD and 84%: ND) and moderate specificities (67%: MD and 62%: ND). After dividing patients into two groups based on the median of PM scores (Fig. 3a, Supplementary Fig. S2A), we drew K-M plots for all patients and for the patients at each stage with MD panel (Fig. 3b–f) and ND panel (Supplementary Fig. S2B–F). Results clearly showed different DFS prognosis between low- and high-risk subtypes at each clinical stage (HRs; Stage 1b: 0.23, Stage 2: 0.22, Stage 3: 0.38, Stage 4: 0, All: 0.43 in MD panel and Stage 1b: 0.34, Stage 2: 0.34, Stage 3: 0.17, Stage 4: 0, All: 0.40 in ND panel). Of the 16 protein biomarkers in the prognosis modules, eight proteins (CATC, CATD, FA11 and ROBO4 in the MD panel and C1QA, CO5, CO7 and CO9 in the ND panel) showed statistical difference between high- and low- risk groups (P < 0.05). The level of these proteins was relatively higher in the higher TNM stage patients, but were not significantly related to Laurens subtype or WHO histology (Supplementary Fig. S3).

**Table 2:** Information of SEPROGADIC-prognosis modules in two independent panels.

MD panel					ND panel				
ID	Coef	SE	P	HR	ID	Coef	SE	P	HR
Stage	0.207	0.051	<0.001	1.23	Stage	0.194	0.051	<0.001	1.21
CATC	−0.299	0.127	0.019	0.74	C1QA	−0.211	0.107	0.051	0.81
CATD	0.512	0.116	<0.001	1.67	CO5	0.306	0.135	0.025	1.36
CD166	−0.324	0.119	0.007	0.72	CO7	0.313	0.138	0.025	1.37
FA10	−0.181	0.143	0.205	0.83	CO9	0.152	0.082	0.068	1.16
FA11	0.244	0.101	0.015	1.28	FBLN1	−0.352	0.132	0.009	0.70
IBP7	−0.072	0.083	0.383	0.93	THRB	−0.214	0.136	0.119	0.81
NCAM1	−0.126	0.102	0.215	0.88					
PLSL	0.172	0.108	0.110	1.19					
ROBO4	0.213	0.100	0.033	1.24					
TRML1	−0.106	0.059	0.074	0.90					

ID: variables (stage and protein names) making up the Cox proportional hazard regression model, Coef: coefficients, SE: standard error, P: P-value, HR: hazard ratio.

To compare the two modules built on ND and MD panels, we divided patients into four groups based on median cutoff values of the two modules and then drew K-M plots (Supplementary Fig. S4). Spearman’s rank correlation between ND and MD modules was 0.456 (P-value < 0.001) and the consistency rate predicting the same prognostic group based on the median threshold value was 67.4% (155 out of 227 samples). The remaining 72 patients were predicted differently by the two modules. The patient group predicted to be high-risk group by both modules had significantly worse prognosis (log-rank test: P < 0.001) than other groups. Even though there is a room to modify the cutoff value, it is clear that our SEPROGADIC-prognosis module can classify poor prognosis group properly.

### SEPROGADIC – AT selection module

**Figure 4:**
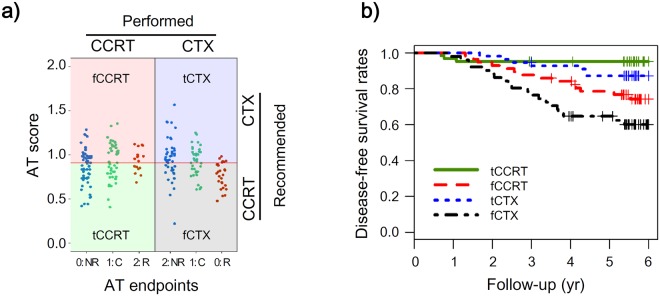
Kaplan-Meier plots of MD panel in SEPROGADIC-AT selection module. (**a**) AT module scores at the three endpoints in the CCRT group (n = 120) and CTX group (n = 107). Patients are divided into four subgroups (true CCRT, false CCRT, true CTX and false CTX) based on the median of AT scores. NR: no recurrence, C: censored, R: recurrence. (**b**) K-M plots of four subtypes. True CCRT (n = 63; observed: 3), false CCRT (n = 57; observed: 14), true CTX (n = 56; observed: 7), false CTX (n = 51; observed: 20).

In the previous ARTIST trial, enrolled GC patients were prescribed with one of CTX or CCRT after surgical resection of tumors. Selection of AT were randomized between patients, and similar number of patients at each tumor stage were treated with either CTX or CCRT. Based on quantitative values of patients’ serum biomarkers, we tried to find a subset of patients with specific characteristics who would benefit from one of CTX or CCRT. To test this assumption, we defined a new endpoint table (detailed in Methods) based on a hypothesis that high-risk patients treated by CTX would have been better if they received CCRT. Inversely, low-risk patients treated by CTX would have been worse if they received CCRT (Fig. 4a). Following the above feature selection and model-building, we constructed SEPROGADIC - AT selection module for both MD and ND panels (Table 3). Based on the median cutoff value, we classified good or poor prognostic groups depending on AT (Fig. 4a and Supplementary Fig. S5A). Patients treated by CCRT with classifier value (AT score; Supplementary Table S6) below the cutoff were defined as true CCRT (tCCRT). Other CCRT patients were defined as false CCRT (fCCRT) in the sense that they had been falsely treated by CCRT. Inversely, CTX patients below the cutoff were defined as false CTX (fCTX) and those above the cutoff were considered as true CTX (tCTX). When we drew K-M plot for all four subtypes (Fig. 4b and Supplementary Fig. S5B), fCTX group showed significantly poor prognosis than the other three groups (HRs: 3.25, tCCRT; 1.50, fCCRT; 2.22, tCTX in MD panel, 2.33, tCCRT; 1.91, fCCRT; 2.37, tCTX in ND panel; P < 0.05). Similarly, prognosis of tCTX showed better prognosis than fCCRT group, especially in MD panel (HR: 0.68; P < 0.05). This suggests that some CTX patients would have shown better prognosis with CCRT instead of CTX.

**Table 3:** Information of SEPROGADIC-AT selection modules in two independent panels.

MD panel				ND panel			
ID	Coef	SE	P	ID	Coef	SE	P
CA2	−0.207	0.092	0.026	C1QA	0.167	0.120	0.165
CADM1	0.408	0.166	0.014	C1QB	0.219	0.150	0.145
CD166	−0.476	0.176	0.007	C1QC	−0.219	0.115	0.058
CO6A1	−0.098	0.105	0.353	FINC	−0.256	0.135	0.060
CYTC	0.443	0.168	0.009	THRB	0.293	0.161	0.071
DPP4	0.138	0.070	0.050	VTDB	−0.267	0.158	0.093
IL1R2	0.234	0.146	0.110				
LAMP1	−0.124	0.109	0.259				
LDHB	0.127	0.136	0.350				
MYG	−0.191	0.099	0.056				
PLSL	−0.296	0.177	0.096				
ROBO4	−0.309	0.118	0.009				
THIO	0.166	0.122	0.173				
TRML1	0.200	0.079	0.013				

ID: variables of Cox regression model, Coef: coefficients, SE: standard error, P: P-value.

## Discussion

Blood biopsy test is a routine procedure in hospital and a non-invasive method for monitoring the health state of disease patients. Biomolecules present in the blood could reflect pathological condition. Serum proteins are promising candidates for capturing the signature of disease. We have developed a serum protein-based prognosis module, SEPROGADIC, to improve TNM staging prediction. Its use during post-surgical treatments will be of great clinical utility. In this study, we adopted two sample preparations before LC-MRM-MS assays: depletion and non-depletion of high abundant proteins for each identical sample. The amount of 14 high abundant proteins was dominant (~94%). Therefore, inclusion of their depletion was an important issue. GC biomarkers in non-depleted whole sera were mostly related to systematic change in body metabolism while GC biomarkers in depleted sera were mostly derived from GC tissue leakage or due to tumor progression^25^.

Abundance of GC biomarkers in 227 patients’ sera is illustrated by heatmaps (Supplementary Fig. S6). In the prognosis module of MD panel, CATD, NCAM1, IBP7, FA11, PLSL, TRML1, CATC, FA10, ROBO4, and CD166 were included. Cathepsin D (CATD) was the best performing variable (AUC: 0.648) out of them. The level of CATD in gastric carcinomas represents poor prognosis^26^. NCAM1 (CD56) is a well-known surface marker of immune cells. Although the origin of serum NCAM1 (CD56) was not defined in the present study, infiltrating CD56+ natural killer T-like cells were reported to be correlated with good prognosis^27^. Co-localization of FA10 with protein Z/protein Z-dependent protease inhibitor complex has been observed in gastric cancer cells, suggesting a role of FA10 in cancer progression^28^. Tissue CD166 is a poor prognosis marker^29^, although the association of serum CD166 with prognosis has not been clearly elucidated yet. There are reports that some other proteins that make up the multi-marker panel or monitored by LC-MRM-MS but excluded from the final panel are also associated with pathogenesis of GC^30–33^. The prognosis module of ND panel includes C1QA, CO5, CO7, FBLN1, THRB, and CO9. Complement components have long been known to be involved in immune response and tumor progression^34^. It has been reported that complement component CO5 is decreased during the first week after surgery but significantly increased after four weeks^35^. Upregulation of CO9 in the plasma of GC patients has been observed by western blot^36^. Complement CO5a/CO5aR pathway potentiates the pathogenesis of GC by down-regulating p21 expression^37^. All these previous reports imply that complement components can affect the prognosis of GC patients. Thus, they were chosen in our prognosis module. The serum biomarkers in our SEPROGADIC worked in tissues of GC patients. When we applied them to mRNA expression level obtained from a publically available GEO dataset (GSE66229)^16^, the prognostic modules outperformed the stage-only models (AUC: 0.783 for MD and 0.751 for ND vs 0.706 for stage-only, Likelihood ratio test: P < 0.001; Supplementary Fig. S7A). Similarly, the biomarkers of AT selection module also worked when applied to GC tissues (Supplementary Fig. S7B). These results show that changes in tumor protein in the blood reflect mRNA changes in tissues of GC.

It has been long known that a radiation therapy directly attacks tumor and indirectly boosts the immune system of patients against tumor^38,39^. Novel therapies in combination with radiation therapy are under development to increase patients’ immune response and reducing toxicity^40^. The final report of ARTIST^6^ showed that there was little difference in the recurrence rate between CCRT and CTX treated patients who received D2 lymph node dissection (P = 0.086). However, CCRT showed better prognosis in lymph node-positive patients (P = 0.0365). In this study, we analyzed serum samples from 227 patients who were part of the same patient group. Our results suggest that we can further refine and divide patients who need to receive CCRT rather than CTX for better prognosis by assaying serum in this patient group of lymph node-positive. This coincides with FDA-recommended concept of companion diagnostics that can improve effects of adjuvant chemotherapy. Since the biomarker proteins included in SEPROGADIC are related to patients’ immune system, lymph node-negative patients may also benefit from adjuvant radiotherapy, and our AT selection module clearly supported this notion.

This study focused on the retrospective analysis of GC patients, and have some limitations such as non-consideration of race heterogeneity, collection of single-center derived samples, and dependency on the ARTIST’s adjuvant therapy regime. The serum markers may have a different tendency depending on the type of drug, dosage, mode of administration used for chemotherapy, and the intensity of radiation therapy. Therefore, it is required to validate this work through future prospective studies. It may also be necessary to analyze SEPROGADIC markers in the patients receiving neoadjuvant therapy or monitor such markers before surgery to stratify the patients and decide treatment modalities. Our prognostic model SEPROGADIC seems to be more complicated than a prediction model using a single biomarker protein. However, it is becoming apparent that a single protein can hardly reflect disease states caused by complex pathologies. In contrast, medical model of multi-marker panel provides a new aspect for clinical intervention into patients. In this regard, there is no doubt that SEPROGADIC can be this kind of model, and it can classify patients into subtypes depending on prognosis and proper treatment.

## Methods

### Study samples

Serum samples were from part of GC patients recruited at Samsung Medical Center (SMC) with informed written consent for the ARTIST trial^6^. Blood were collected after 4 weeks of D2 lymph node dissection before receiving any AT. For blood preparations, 3 mL of blood was collected into an EDTA tube and placed on ice for transport to the laboratory where they were centrifuged, aliquoted, and immediately frozen at −80 °C until use. Sera were prepared as suggested by the Human Proteome Organization (HUPO) Plasma Proteome Project^41^. This study was approved by the Institutional Review Board of Samsung Medical Center. It followed the tenets of the Declaration of Helsinki. The trial was registered at clinicaltrials.gov (Identifier: NCT00323830).

### Sample processing

Serum samples were processed by two different ways. First, 600 fmol of recombinant *Escherichia coli* β-galactosidase protein was spiked into 12 μL of serum. The top fourteen abundant proteins were then depleted using MARS14 (Agilent, CA, USA) column. For this, the mixture was diluted 1:5 with a proprietary “Buffer A” and loaded onto MARS14 column on an Agilent 1100 series HPLC system. Unbound fraction was buffer-exchanged into 8 M urea in 50 mM Tris (pH 8) and concentrated through ultrafiltration using Amicon Ultra-0.5 mL 3 kDa cutoff filter (Millipore, Darmstadt, Germany) to approximately 50 μL (denoted as MD sample). On the other hand, whole serum (1.0 µL) without any depletion process was buffered with 40 µL of 8 M urea in 50 mM Tris (pH 8.0) and spiked with 600 fmol recombinant *E. coli* β-galactosidase (denoted as ND sample). Samples prepared in these two ways were treated with 5 µL of 50 mM TCEP (tris(2-carboxyethyl)phosphine) at 25 °C for 1 hr and further treated with 5 µL of 150 mM iodoacetamide at 25 °C for 1 hr in the dark. Urea concentration was diluted to 4.0 M with 50 mM Tris prior to Lys-C digestion (Wako, Richmond, VA, USA) with enzyme to substrate ratio of 1:100 and incubated at 25 °C for 4 hr with mixing on a shaker at 600 rpm. The sample was further diluted 5-fold with 50 mM Tris (pH 8.0) to bring urea concentration to 0.8 M. Sequencing-grade trypsin (Promega, Madison, WI, USA) was then added to the sample at an enzyme-to-protein ratio of 1:50 and incubated at 37 °C for 12 hr with shaking at 600 rpm. Formic acid was then added to a final concentration of 0.3% to stop the digestion reaction. The peptide mixture was then desalted with a C-18 macrospin column cartridge (Harvard Apparatus, MA, USA), dried with a vacuum centrifuge (miVac Duo Concentrator, Genevac, Suffolk, UK), and stored at −80 °C until use.

### Deep down Profiling of serum proteins by bRPLC-HPLC-MS/MS

Serum samples were mixed in the same ratio without spiking with *E. coli* β-galactosidase. The top 12 abundant proteins were depleted with an affinity spin column (ThermoFisher Scientific, Bremen, Germany). Reduction, alkylation, and digestion were carried as described above. Digested peptides were separated into 96 fractions by basic reversed phase liquid chromatography (bRPLC). Every 12^th^ fraction was collected and mixed separately^42^. The resultant 12 concatenated fractions were vacuum-dried. Dried peptide samples were reconstituted in 0.4% acetic acid and an aliquot containing approximately 1 μg was injected from a cooled (10 °C) autosampler into a reversed-phase Magic C18aq (Michrom BioResources, Auburn, CA, USA) column (15 cm × 75 μm, packed in-house) on an Eksigent nanoLC-ultra 1D plus system at a flow rate of 300 nL/min. Prior to use, the column was equilibrated with 95% buffer A (0.1% formic acid in water) and 5% buffer B (0.1% formic acid in acetonitrile). Peptides were eluted with a linear gradient from 5% to 50% buffer B over 200 min and 50% to 80% buffer B over 5 min followed by an organic wash and aqueous re-equilibration at a flow rate of 300 nL/min with a total run time of 230 min. The HPLC system was coupled to a Q-Exactive mass spectrometer (ThermoFisher Scientific, Bremen, Germany) operated in a data-dependent acquisition (DDA) mode and DDA-Exclusion mode. The DDA-exclusion mode has been well described in our previous paper^43^. MS setting was identical to that used in the published study^43^.

A total of 24 raw files (*.raw; DDA and DDA-Exclusion for 12 fractions) were transformed to mzML files with msConvert program. We used wavelet-based peak picking algorithm [Command: msconvert *.raw–filter “peakPicking cwt 1.2 0.01 2-“–filter “turbocharger”–filter “MS2Deisotope Poisson”]^44^. Database search was performed using MSGF+ percolator (MSGF+ ; version43)^45^ against human UniprotKB-SwissProt database (released 2015.03.04) at peptide identification FDR < 0.01. Search options used were: profile mode, number of allowed modifications = 4, fixed modification of carbarmidomethylation at cysteine = 57.0215, and optional modification of oxidation at methionine = 15.9949.

### Selection of target peptides and MRM optimization

We considered 284 GC biomarker candidates. From the reference UniProt database, we gathered unique peptides for candidate proteins with the following criteria: (1) consisting of 6–20 amino acids, (2) having no missed cleavage site (i.e. no internal lysine or arginine), and (3) containing none of methionine, N-terminal glutamine, known single amino acid polymorphism, or post-translational modification. The above criteria allowed us to select 589 peptides. These peptides were synthesized (SpikeTides, JPT Peptide Technologies GmbH, Germany) and used for optimization of MRM parameters: collisional energy (CE), declustering potential (DP), and cell exit potential (CXP). The optimization was performed by direct infusion of synthesized peptides to Qtrap5500 using Turbospray (SCIEX, Foster City, CA, USA) (Supplementary Table S1).

### LC-MRM-MS

The LC system was comprised of an Ekisigent nanoLC-Ultra 2D plus coupled with a NanoFlex system (SCIEX, Foster City, CA, USA). Mobile phase A was 0.1% formic acid in water and mobile phase B was 0.1% formic acid in acetonitrile. Peptide samples were reconstituted with 22.5 µL of 2% mobile phase B, injected with a full sample loop injection of 1 μL, and separated on a reversed-phase column packed with ReproSil-Pur C18 resin (75 µm i.d., 12 cm length, pore size 120 Å, particle size 3 µm; packed in-house). The column was priory equilibrated with 5% mobile phase. Peptides were eluted at a flow rate of 350 nL/min. Elution conditions were slightly different depending on the type of samples (MD vs. ND) because MD panel included more peptides to be monitored than ND panel. For ND samples, the gradient of %B was 5–30–50–50–5–5% for 30, 1, 7, 1, and 5 min at each interval. For MD samples, it was 5–20–35–50–50–5–5% for 40, 10, 1, 10, 1, and 12 min. After peptide elution, the column was washed again with 50% B for 30 min and re-equilibrated with the initial condition for 30 min.

The LC system was coupled to a Qtrap5500 mass spectrometer via a nanoelectrospray ion source (SCIEX, Foster City, CA, USA). MS detection was carried out in positive MRM mode with the following parameters: ion spray voltage of 2200 V, curtain gas at 25 psi, ion source gas at 40 psi, resolution at 0.7 Da (unit resolution) for Q1/Q3, interface temperature at 150 °C, and scan mass range of m/z > 300–1250. MRM experiments were performed using 5-min scheduled MRM mode with less than 110 concurrent transitions for ND samples and 10-min scheduled MRM mode with less than 200 concurrent transitions for MD samples. The mass spectrometer was operated with Analyst software (Version 1.5.2, SCIEX) which generated MRM-MS data (*.wiff).

Samples were analyzed batch wise. All samples in one batch were continuously analyzed without interruption. The entire 227 serum samples were divided into 10 batches. Each batch contained one quality control sample (a standard sample made by mixing all entire sera in the same ratio) and 21–24 clinical samples. From each batch, two samples were randomly selected and measured three times. Therefore, 1 + 19 to 22 + 3 + 3 LC-MRM-MS data were generated from a batch. Skyline (version 2.6.0)^46^ was used to analyze MRM results from extracted ion chromatogram (XIC). Raw chromatograms of all transitions of peptides were manually reviewed using the software. For each peptide, we determined a single quantitative transition which had the least interference and the most sensitivity. Other transitions were used for peak assignment only. These raw data were deposited in PASSEL database (accession ID, PASS01140)^47^.

### Selection of endogenous normalizing proteins

Raw LC-MRM-MS data were normalized with endogenous normalizing proteins to reduce the effect of instrumental response variation over time. High abundant plasma proteins in the range of tens to hundreds μg/mL were primary candidates of normalizing proteins. From the Plasma Proteome Database (PPD), we gathered peptides consistent with conditions of MRM target selection and annotated as ‘MRM detection’^20^. There were 35 peptides of 35 proteins. MRM transitions and MRM energy parameters were obtained from SRMAtlas (http://www.srmatlas.org/) in peptideAtlas and used as default values in Skyline software. One representative peptide per protein for a total of 15 proteins was monitored during LC-MRM-MS. Five out of these fifteen were finally selected as suitable for MRM normalization based on the following criteria: (1) detected in all samples and in both sample preparations of MD and ND; (2) their serum levels were not significantly different among three patient groups consisting of patients recurred within 6 years (n = 44), patients censored before 6 years (n = 80), and patients without recurrence until 6 years (n = 103) as proved by ANOVA test (p > 0.05) or disease free survival analysis; (3) their serum level was highly correlated with exogenously spiked β-galactosidase as determined by Pearson correlation coefficient > 0.5 between average peak areas of β-galactosidase peptides and raw peak areas of normalizing peptides; (4) had nearly constant serum concentration throughout the sample as top-ranked by NormFinder stability value^21^.

### Normalization of raw LC-MRM-MS data

Raw peak areas of the selected five normalizing proteins (more specifically, MRM transitions) in each sample were divided by the corresponding median value of all samples. And then, the median of five ratios of the sample was used as the normalization scaling factor (NSF) for that sample. NSF for sample *s* is given by the following equation:

$$NS{F}_{s}=median(\frac{{N}_{1,s}}{{\widehat{N}}_{1}},\frac{{N}_{2,s}}{{\widehat{N}}_{2}},\ldots ,\frac{{N}_{5,s}}{{\widehat{N}}_{5}})$$

where ${N}_{i,s}$ is the raw peak area of a normalization transition *i* in sample *s* and ${\widehat{N}}_{i}$ is the median of the peak area of the transition *i* in the entire sample. For each transition of biomarker candidates in a sample, its normalized peak area was calculated by dividing its raw peak area by NSF.where  is the normalized peak area of *j*-th biomarker candidate in sample *s* and $P{A}_{j,s}$ is the raw peak area of the corresponding transition.

The use of NSF assumes that instrumental response variation affects all peptide ions to the same extent which is almost correct, but not always. The ionization efficiency of each peptide depends on the amount of whole ionized peptides, and it varies depending on the nature of the target peptide. We denoted this as ${{\rm{\beta }}}_{j}$ and drew this value from the slope in a plot of ${{\rm{l}}{\rm{o}}{\rm{g}}}_{2}(P{A}_{j,s})\,$versus ${\mathrm{log}}_{2}(NS{F}_{s})$ of ten quality control samples. ${{\rm{\beta }}}_{j}$ was very close to one, as expected. β-corrected normalized peak area was calculated using the following equation:

### Feature selection for multi-marker panel in multivariate Cox regression analysis

Eight-fold cross-validation was applied to select the most valuable protein variables for prognostic and AT selection modules. Considering two panels for each module, the total number of clinical cox regression models was four. In prognostic modules, patients were divided into three groups according to prognostic outcome and a PM endpoint was given to each group: without recurrence until 6-year (PM endpoint = 0), censored before 6-year (PM endpoint = 1), and recurred within 6-year (PM endpoint = 2)^48^. On the other hand, we designed a new definition system for AT outcome (AT endpoint) to build the AT selection module. AT endpoints of the CCRT-treated patient group were the same as PM endpoints. However, AT endpoints of the CTX-treated patient group were given in the reverse order of PM endpoints (for example, AT endpoint of 2 was given to CTX-treated patient group without recurrence until 6-year).

Each cross-validation was performed with backward elimination at a P-value cutoff of 0.05. The 8-fold cross validation was repeated 500 times. In case of a prognosis module, clinical stage value was a forced variable. At each iteration, we observed whether a protein was selected at least four times out of eight. If so, we increased the number of observations by one. After 500 repeated validation process, we arranged protein features in the descending order of the observation number and confirmed that proteins from the first protein to a specific protein were selected at a higher probability than when they were randomly extracted. When randomly selecting *s* from *n* features (ND: 20, MD: 73), the probability of a particular feature coming out four or more times out of eight is given according to a binary distribution:

$${p}_{s}={\sum }_{i=4}^{8}(\begin{array}{c}8\\ i\end{array})\,\ast \,\,{(\frac{s}{n})}^{i}\,\ast \,\,{(\frac{(n-s)}{n})}^{8-i}$$

Since we repeated the cross validation 500 times, we selected protein features from the first feature to the *s*-th feature satisfying the following Z-statistic

$$Z=\frac{({\rm{N}}.{\rm{O}}.({\rm{s}})-500\,\ast \,\,{p}_{s})}{\sqrt{500\,\ast \,\,{p}_{s}\,\ast \,\,(1-\,{p}_{s})}}\ge 1.96\,({\rm{confidence}}\,{\rm{level}}\,97.5 \% )$$

where N.O.(s) is the number of observation of *s*-th feature.

### Analysis of public microarray data

We downloaded the gene expression profile data (series accession number: GSE66229) in the Gene Expession Omnibus database^49^. From the 300 patient microarray data, we excluded 30 data with long-distance metastasis, no AT information, or uncertain TNM stage information, and used only 270 data. The platform used was GPL570 (Affymetrix Human Genome U133 Plus 2.0 Array; Agilent Technologies, Palo Alto, CA, USA). Using preprocessCore package in R (http://www.bioconductor.org/packages/3.0/bioc/html/preprocessCore.html), quantile normalization was performed to obtain standardized microarray data. Matching probes to gen symbols based on the annotation information of platform GPL570 by Affy package in R. Subsequently, the gene expression level of each gene was calculated by determining the highest expression levels of probes corresponding to the same gene.

### Statistical analyses

Kaplan-Meier analysis was performed using MedCalc (version 14.12) for Windows. A survivalROC analysis was performed with RStudio (version 0.98.953) including R (version 3.4.2). Other software packages included boot for calculating the confidence interval of AUC values, ggplot2 for drawing boxplots and ROC curves, and Heatplus for drawing heatmaps.

## Electronic supplementary material

Supplementary Information

Supplementary Table S1

Supplementary Table S2

Supplementary Table S3

Supplementary Table S4

Supplementary Table S5

Supplementary Table S6

## References

[1] Global Burden of Disease Cancer Collaboratio*n et al*. Global, Regional, and National Cancer Incidence, Mortality, Years of Life Lost, Years Lived With Disability, and Disability-Adjusted Life-years for 32 Cancer Groups, 1990 to 2015: A Systematic Analysis for the Global Burden of Disease Study. *JAMA Onco*l **3**, 524–548, 10.1001/jamaoncol.2016.5688 (2017). 10.1001/jamaoncol.2016.5688 PMC6103527 27918777

[2] Agolli L, Maurizi Enrici R, Osti MF (2016). Adjuvant radiochemotherapy for gastric cancer: Should we use prognostic factors to select patients?. World J Gastroenterol.

[3] Macdonald JS (2001). Chemoradiotherapy after surgery compared with surgery alone for adenocarcinoma of the stomach or gastroesophageal junction. N Engl J Med.

[4] Cunningham D (2006). Perioperative chemotherapy versus surgery alone for resectable gastroesophageal cancer. N Engl J Med.

[5] Kim TH (2012). Phase 3 trial of postoperative chemotherapy alone versus chemoradiation therapy in stage III-IV gastric cancer treated with R0 gastrectomy and D2 lymph node dissection. Int J Radiat Oncol Biol Phys.

[6] Lee J (2012). Phase III trial comparing capecitabine plus cisplatin versus capecitabine plus cisplatin with concurrent capecitabine radiotherapy in completely resected gastric cancer with D2 lymph node dissection: the ARTIST trial. J Clin Oncol.

[7] Sano T (2004). Gastric cancer surgery: morbidity and mortality results from a prospective randomized controlled trial comparing D2 and extended para-aortic lymphadenectomy–Japan Clinical Oncology Group study 9501. J Clin Oncol.

[8] Hartgrink HH (2004). Extended lymph node dissection for gastric cancer: who may benefit? Final results of the randomized Dutch gastric cancer group trial. J Clin Oncol.

[9] Papadopoulos N, Kinzler KW, Vogelstein B (2006). The role of companion diagnostics in the development and use of mutation-targeted cancer therapies. Nat Biotechnol.

[10] Jorgensen JT (2015). Clinical application of companion diagnostics. Trends Mol Med.

[11] Geyer PE, Holdt LM, Teupser D, Mann M (2017). Revisiting biomarker discovery by plasma proteomics. Mol Syst Biol.

[12] Rifai N, Gillette MA, Carr SA (2006). Protein biomarker discovery and validation: the long and uncertain path to clinical utility. Nat Biotechnol.

[13] Park JM (2015). Integrated analysis of global proteome, phosphoproteome, and glycoproteome enables complementary interpretation of disease-related protein networks. Sci Rep.

[14] Marimuthu A (2013). SILAC-based quantitative proteomic analysis of gastric cancer secretome. Proteomics Clin Appl.

[15] Subbannayya Y (2015). Identification of differentially expressed serum proteins in gastric adenocarcinoma. J Proteomics.

[16] Cristescu R (2015). Molecular analysis of gastric cancer identifies subtypes associated with distinct clinical outcomes. Nat Med.

[17] Surinova S (2011). On the development of plasma protein biomarkers. J Proteome Res.

[18] Kusebauch U (2016). Human SRMAtlas: A Resource of Targeted Assays to Quantify the Complete Human Proteome. Cell.

[19] Deutsch EW (2010). The PeptideAtlas Project. Methods Mol Biol.

[20] Nanjappa V (2014). Plasma Proteome Database as a resource for proteomics research: 2014 update. Nucleic Acids Res.

[21] Andersen CL, Jensen JL, Orntoft TF (2004). Normalization of real-time quantitative reverse transcription-PCR data: a model-based variance estimation approach to identify genes suited for normalization, applied to bladder and colon cancer data sets. Cancer Res.

[22] Heagerty PJ, Zheng Y (2005). Survival model predictive accuracy and ROC curves. Biometrics.

[23] Heagerty PJ, Lumley T, Pepe MS (2000). Time-dependent ROC curves for censored survival data and a diagnostic marker. Biometrics.

[24] Pourhoseingholi MA, Baghestani AR, Vahedi M (2012). How to control confounding effects by statistical analysis. Gastroenterol Hepatol Bed Bench.

[25] Anderson NL, Anderson NG (2002). The human plasma proteome: history, character, and diagnostic prospects. Mol Cell Proteomics.

[26] Manuel Del Casar J (2004). Prognostic value of cytosolyc cathepsin D content in resectable gastric cancer. J Surg Oncol.

[27] Peng LS (2016). Altered phenotypic and functional characteristics of CD3+ CD56+ NKT-like cells in human gastric cancer. Oncotarget.

[28] Sierko E (2014). Protein Z/protein Z-dependent protease inhibitor system in loco in human gastric cancer. Ann Hematol.

[29] Ishigami S (2011). Clinical implication of CD166 expression in gastric cancer. J Surg Oncol.

[30] Gofuku J (1998). Characterization of soluble E-cadherin as a disease marker in gastric cancer patients. Br J Cancer.

[31] Kawamura J (2007). Clinicopathological significance of aminopeptidase N/CD13 expression in human gastric carcinoma. Hepatogastroenterology.

[32] Stabuc B, Vrhovec L, Stabuc-Silih M, Cizej TE (2000). Improved prediction of decreased creatinine clearance by serum cystatin C: use in cancer patients before and during chemotherapy. Clin Chem.

[33] Hu X, Huang Z, Liao Z, He C, Fang X (2014). Low CA II expression is associated with tumor aggressiveness and poor prognosis in gastric cancer patients. Int J Clin Exp Pathol.

[34] Pio R, Corrales L, Lambris JD (2014). The role of complement in tumor growth. Adv Exp Med Biol.

[35] Suarez J (1984). Immunologic responses following surgical resection of gastrointestinal carcinomas. Rev Esp Oncol.

[36] Chong PK (2010). Upregulation of plasma C9 protein in gastric cancer patients. Proteomics.

[37] Chen J (2018). Complement C5a/C5aR pathway potentiates the pathogenesis of gastric cancer by down-regulating p21 expression. Cancer Lett.

[38] Baird JR (2017). Stimulating Innate Immunity to Enhance Radiation Therapy-Induced Tumor Control. Int J Radiat Oncol Biol Phys.

[39] Hu ZI, Ho AY, McArthur HL (2017). Combined Radiation Therapy and Immune Checkpoint Blockade Therapy for Breast Cancer. Int J Radiat Oncol Biol Phys.

[40] He C (2016). Core-shell nanoscale coordination polymers combine chemotherapy and photodynamic therapy to potentiate checkpoint blockade cancer immunotherapy. Nat Commun.

[41] Rai AJ (2005). HUPO Plasma Proteome Project specimen collection and handling: towards the standardization of parameters for plasma proteome samples. Proteomics.

[42] Kim JS (2014). Detection and quantification of plasma amyloid-beta by selected reaction monitoring mass spectrometry. Anal Chim Acta.

[43] Yeom J, Kabir MH, Lee C (2015). Impact of data-dependent exclusion list based mass spectrometry on label-free proteomic quantification. Rapid Commun Mass Spectrom.

[44] French WR (2015). Wavelet-based peak detection and a new charge inference procedure for MS/MS implemented in ProteoWizard’s msConvert. J Proteome Res.

[45] Granholm V (2014). Fast and accurate database searches with MS-GF+ Percolator. J Proteome Res.

[46] MacLean B (2010). Skyline: an open source document editor for creating and analyzing targeted proteomics experiments. Bioinformatics.

[47] Farrah T (2012). PASSEL: the PeptideAtlas SRMexperiment library. Proteomics.

[48] Surinova S (2015). Non-invasive prognostic protein biomarker signatures associated with colorectal cancer. EMBO Mol Med.

[49] Barrett T, Edgar R (2006). Gene expression omnibus: microarray data storage, submission, retrieval, and analysis. Methods Enzymol.

