# Supplementary material for: SEPROGADIC – serum protein-based gastric cancer prediction model for prognosis and selection of proper adjuvant therapy
Source: Sci Rep. 2018 Nov 15;8:16892. doi: 10.1038/s41598-018-34858-x (PMC6237900; doi:10.1038/s41598-018-34858-x)
Supplement: Supplementary file 1 — Supplementary Information [file 41598_2018_34858_MOESM1_ESM.pdf]

Supplementary Information

SEPROGADIC – serum protein-based gastric cancer prediction model for prognosis and selection of proper adjuvant therapy

Hee-Sung Ahn[1,2], Tae Sung Sohn[3], Mi Jeong Kim[1], Byoung Kyu Cho[4], Su Mi Kim[3], Seung Tae Kim[5], Eugene C. Yi[4], Cheolju Lee[1,2,6,*]

[1] Center for Theragnosis, Korea Institute of Science and Technology, 5 Hwarangro-14-gil, Seongbuk-gu, Seoul 02792, Republic of Korea

[2] Division of Bio-Medical Science & Technology, KIST School, Korea University of Science and Technology, 5 Hwarangro-14-gil, Seongbuk-gu,Seoul 02792, Republic of Korea

[3] Department of Surgery, Samsung Medical Center, Sungkyunkwan University School of Medicine, 81 Irwon-ro, Gangnam-gu, Seoul 06351, Republic of Korea

[4] Department of Molecular Medicine and Biopharmaceutical Sciences, School of Convergence Science and Technology and College of Medicine, Seoul National University, 103 Daehak-ro, Jongno-gu, Seoul 03080, Republic of Korea

[5] Department of Medicine, Samsung Medical Center, Sungkyunkwan University School of Medicine, 81 Irwon-ro, Gangnam-gu, Seoul 06351, Republic of Korea

[6] KHU-KIST Department of Converging Science and Technology, Kyung Hee University, 26 Kyunghee-daero, Dongdaemun-gu, Seoul 02447, Republic of Korea

*Corresponding author

Contact Information

clee270@kist.re.kr

Keywords

gastric cancer; prognosis; adjuvant therapy; multiple reaction monitoring; clinical model

Supplementary Figures

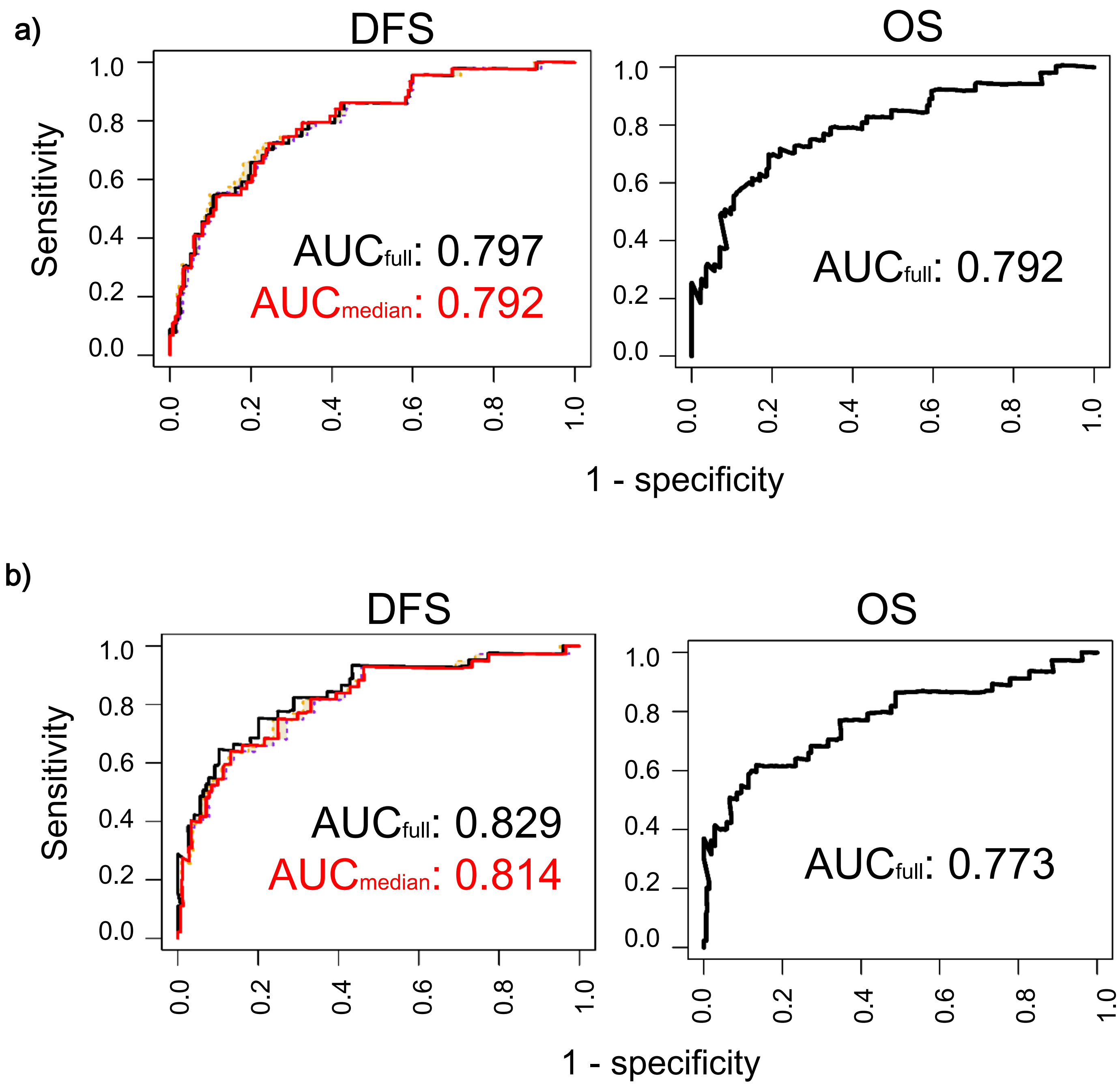

Supplementary Figure S1. Survival ROC analysis of 6-year disease-free-survival (DFS) and overall survival (OS). (a) Two ROC curves based on ten proteins (CATC, CATD, CD166, FA10, FA11, IBP7, NCAM1, PLSL, ROBO4, and TRML1) and stage in MD panel (median AUC: 0.792; full AUC: 0.797 and full AUC: 0.792; OS). (b) Two ROC curves based on six proteins (C1QA, CO5, CO7, CO9, FBLN1, and THRB) and stage in ND panel (median AUC: 0.814; full AUC: 0.829; DFS and full AUC: 0.773; OS). We implemented 5-fold cross validation analysis testing through 2001 times bootstrapping (full dataset consensus model is colored in black, median in red, 25th percentile in magenta, and 75th percentile in orange).

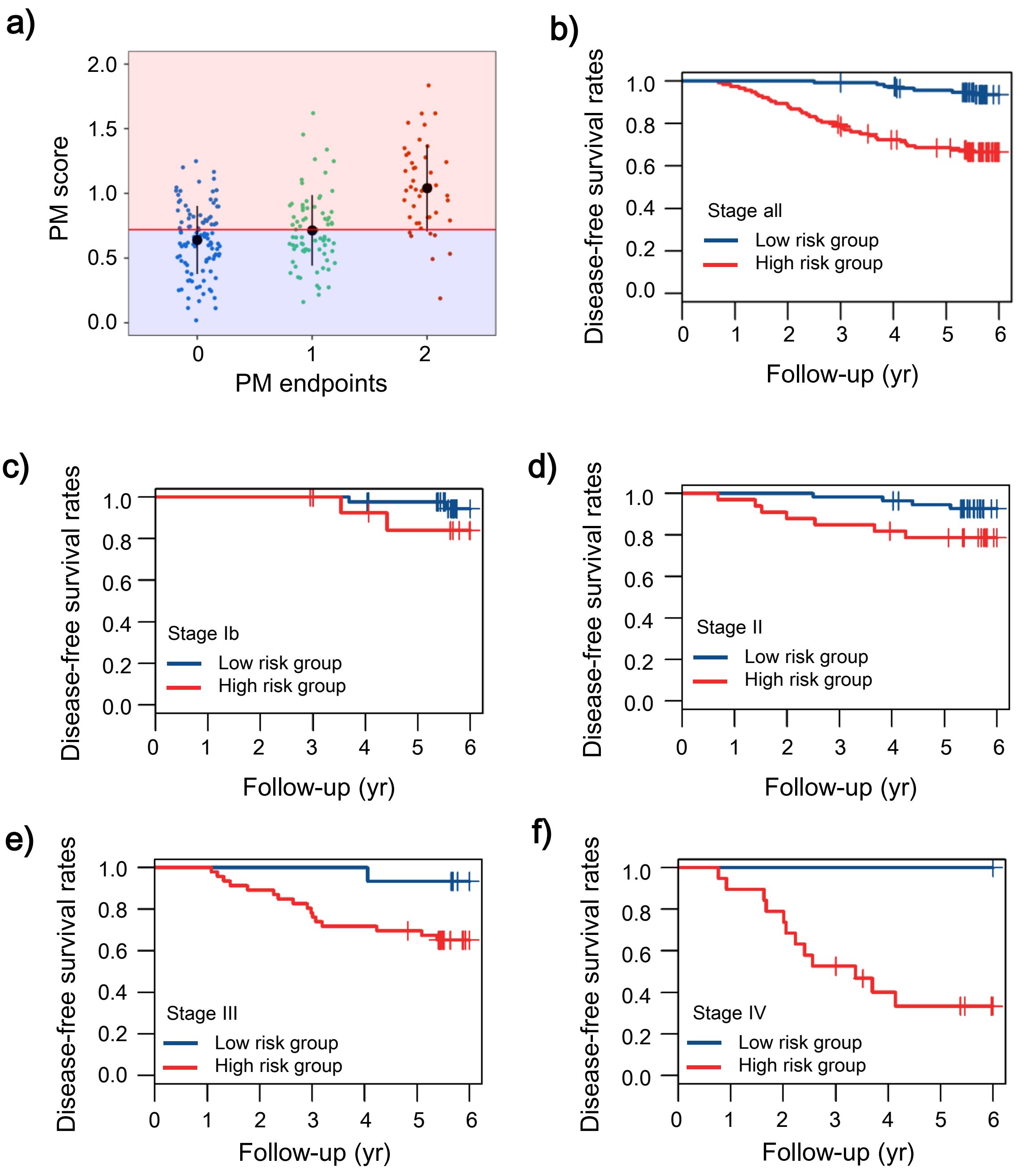

Supplementary Figure S2. Kaplan-Meier plots of ND panel in SEPROGADIC prognostic module. We divided patients at each stage into two risk groups by median outcome value. (a) Classification of all the patients into two risk groups by the median of prognostic module (PM) scores. (b) Stage all: low risk group (n = 114; observed: 7), high risk group (n = 113; observed: 37), P = 1e-07. (c) Stage 1b: low risk group (n = 43; observed: 2), high risk group (n = 15; observed: 2), P = 0.204. (d) Stage 2: low risk group (n = 55; observed: 4), high risk group (n = 33; observed: 7), P = 0.044. (e) Stage 3: low risk group (n = 15; observed: 1), high risk group (n = 46; observed: 16), P = 0.04. (f) Stage 4: low risk group (n = 1; observed: 0), high risk group (n = 19; observed: 12), P = 0.308.

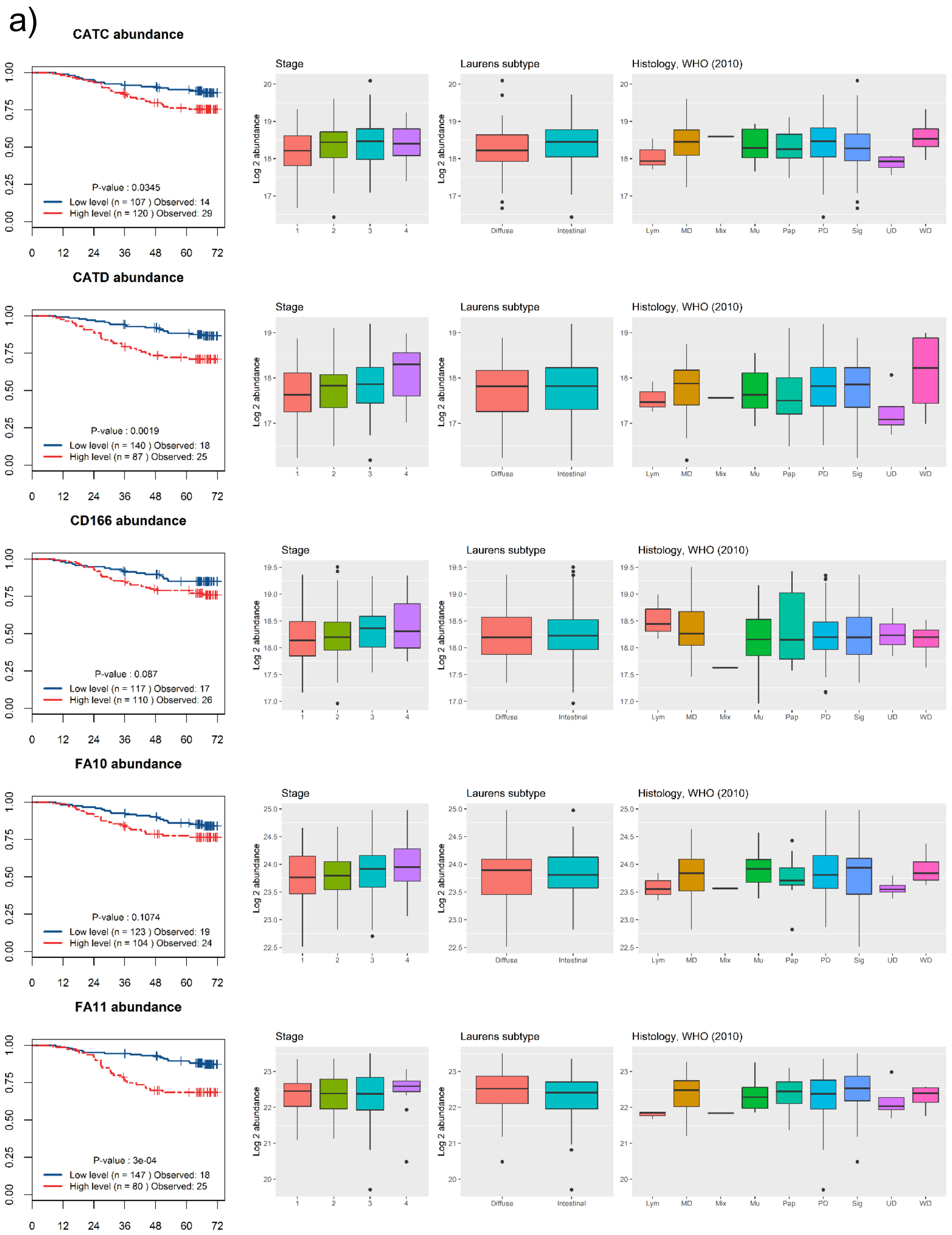

Histology, WHO (2010):
Mucinous adenocarcinoma (Mu; n = 11), Signet-ring cell carcinoma (Sig; n = 66),
Moderately differentiated (MD; n = 51), Poorly differentiated (PD; n = 77),
Undifferentiated carcinoma (UD; n = 4), Lymphoepitheliomatous carcinoma (Lym; n = 3),
Papillary adenocarcinoma (Pap; n = 10), Well differentiated (WD; n = 4),
Mixed adeno-neuroendocrine carcinoma (Mix; n = 1)

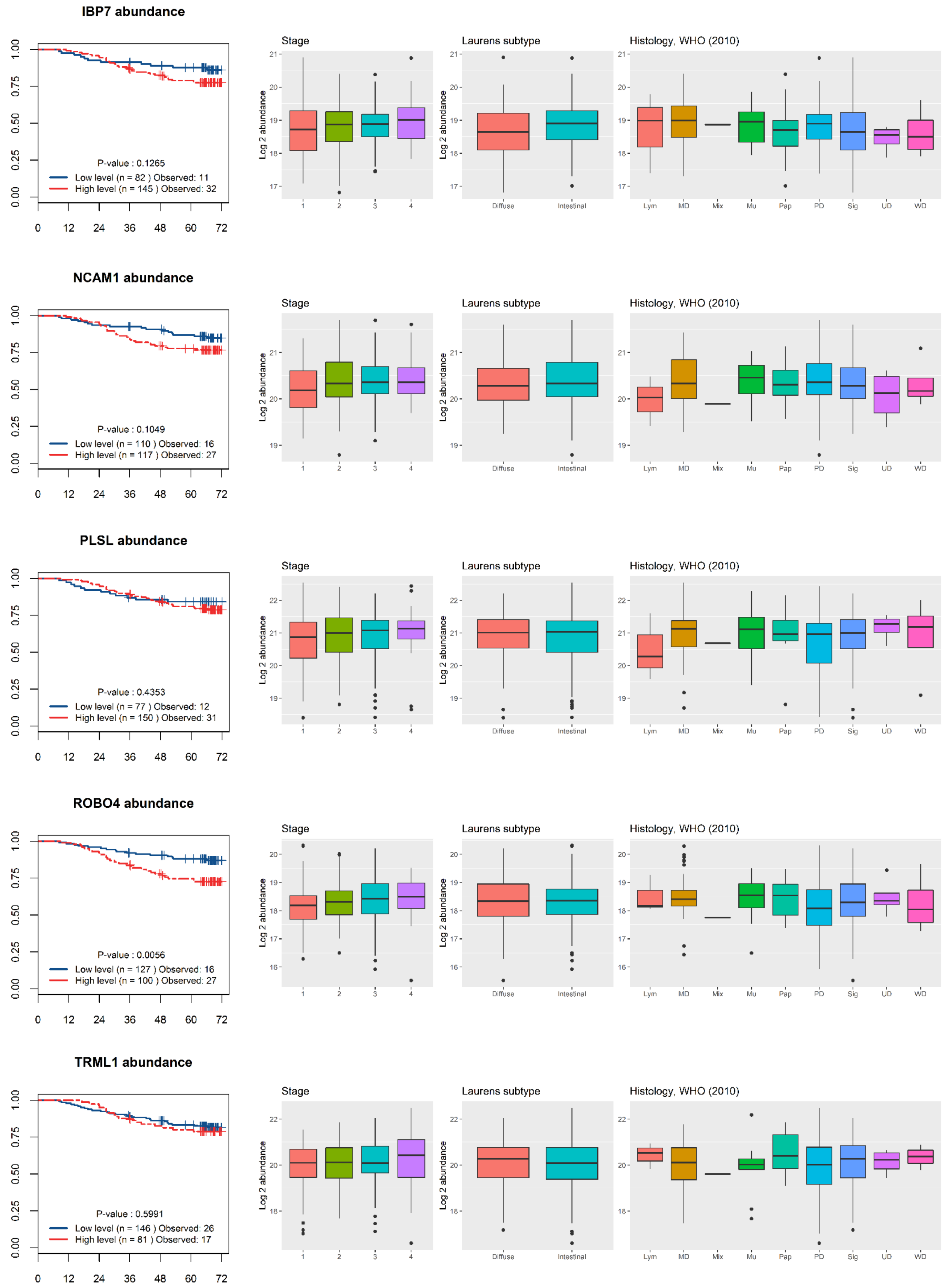
IBP7 abundance
P-value : 0.1265
Low level (n = 82) Observed: 11
High level (n = 145) Observed: 32
NCAM1 abundance
P-value : 0.1049
Low level (n = 110) Observed: 16
High level (n = 117) Observed: 27
PLSL abundance
P-value : 0.4353
Low level (n = 77) Observed: 12
High level (n = 150) Observed: 31
ROBO4 abundance
P-value : 0.0056
Low level (n = 127) Observed: 16
High level (n = 100) Observed: 27
TRML1 abundance
P-value : 0.5991
Low level (n = 146) Observed: 26
High level (n = 81) Observed: 17
Stage
Laurens subtype
Histology, WHO (2010)
Log 2 abundance
Diffuse
Intestinal
Lym
MD
Mix
Mu
Pap
PD
Sig
UD
WD

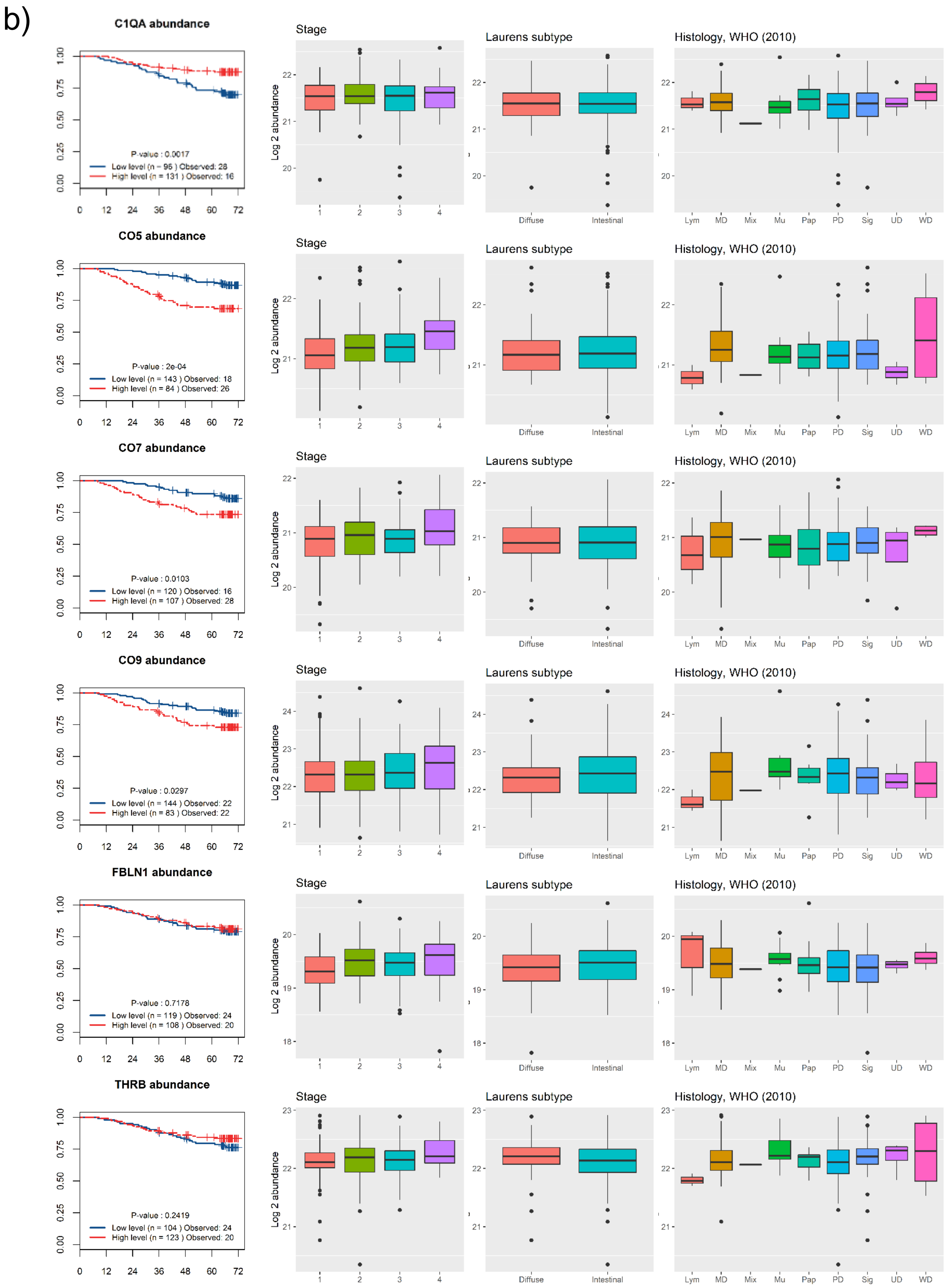

Supplementary Figure S3. K-M plot, boxplots of stage, Laurens subtype and WHO histology for prognostic biomarkers (a) 10 proteins and 227 samples (MD panel) and (b) 6 proteins and 227 samples (ND panel).

a)

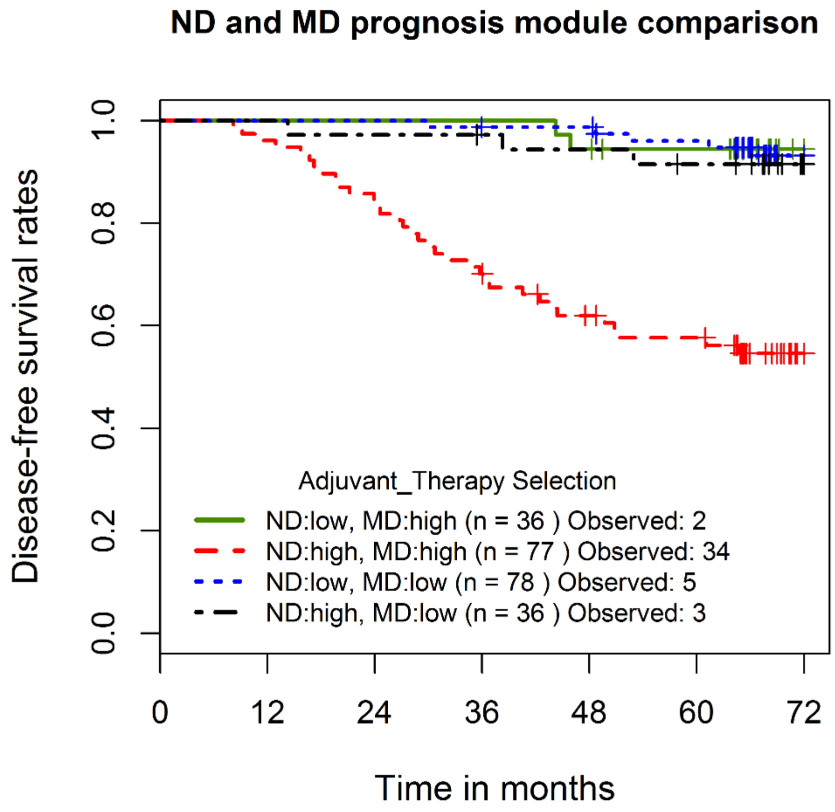
ND and MD prognosis module comparison
Disease-free survival rates
1.0
0.8
0.6
0.4
0.2
0.0
0
12
24
36
48
60
72
Time in months
Adjuvant_Therapy Selection
ND:low, MD:high (n = 36) Observed: 2
ND:high, MD:high (n = 77) Observed: 34
ND:low, MD:low (n = 78) Observed: 5
ND:high, MD:low (n = 36) Observed: 3

b)

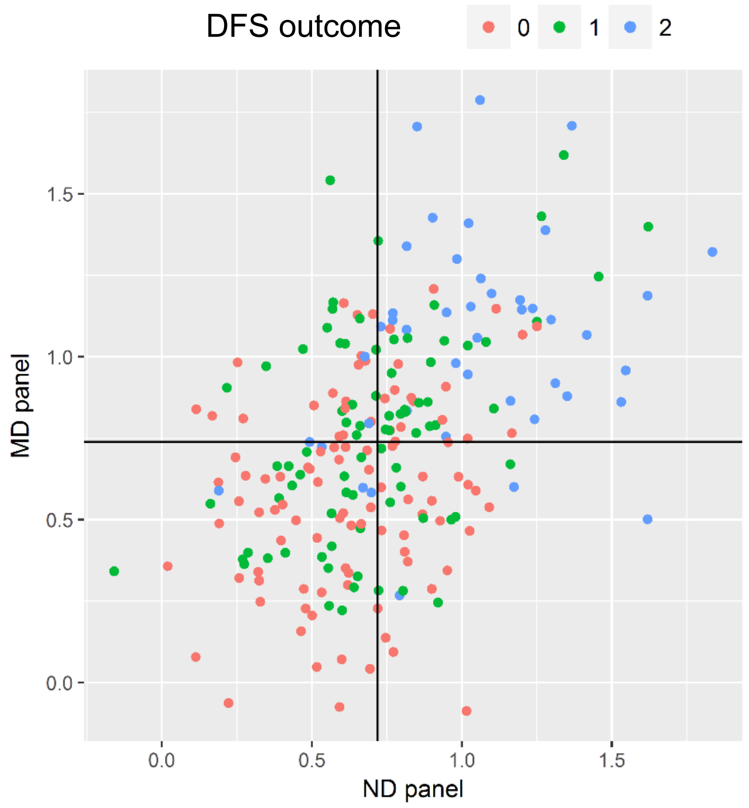
DFS outcome
0
1
2
MD panel
1.5
1.0
0.5
0.0
ND panel
0.0
0.5
1.0
1.5

c)

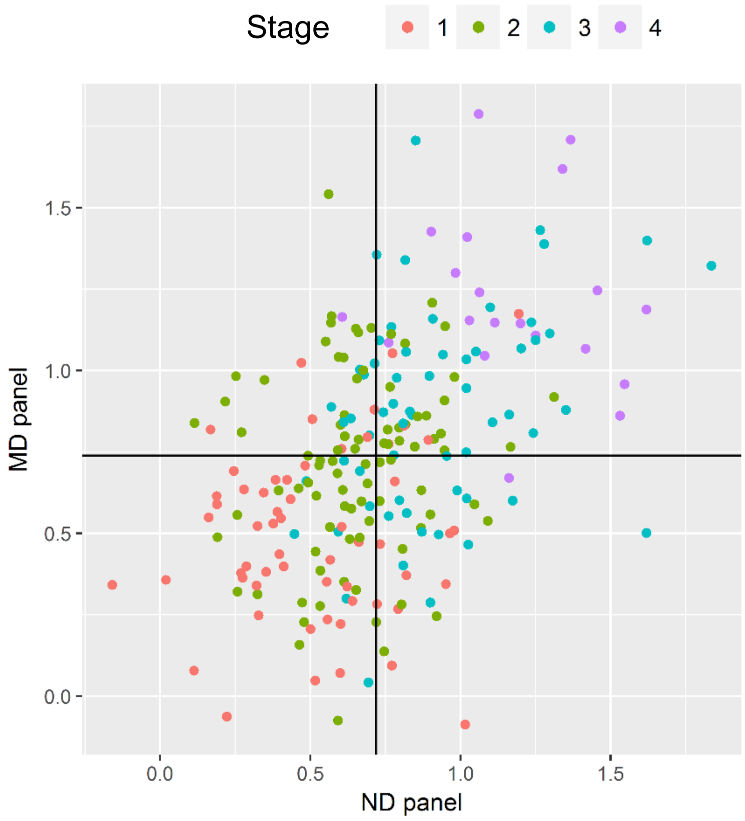

d)

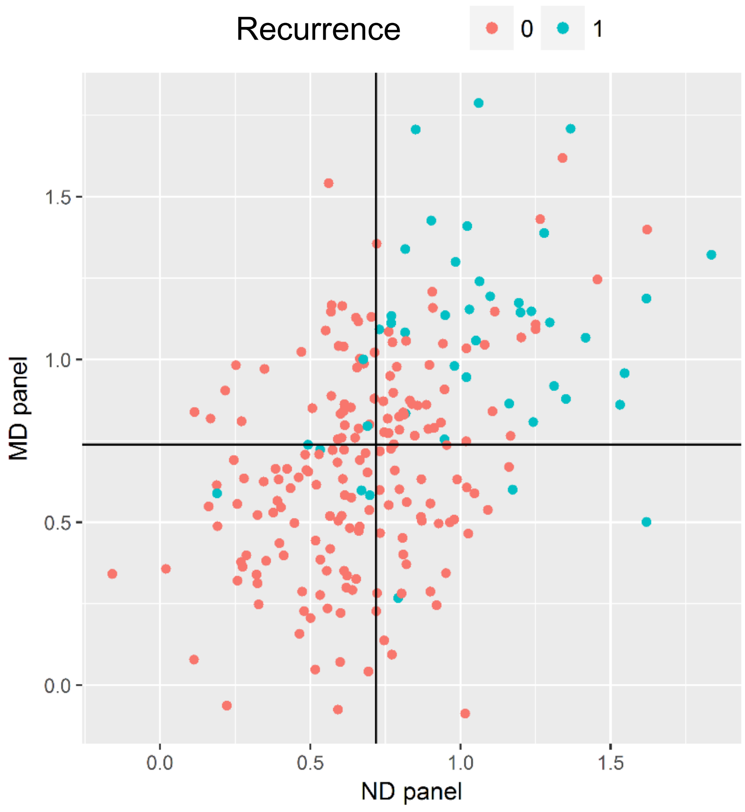

Supplementary Figure S4. Consistency of 227 prognostic values between ND and MD panels. (a) K-M plot of four different risk groups – high and low risk groups based on ND and MD prognosis models. (b-d) scatter plots (b) DFS outcome, (c) stage and (d) recurrence of high and low risk groups based on ND and MD prognosis models.

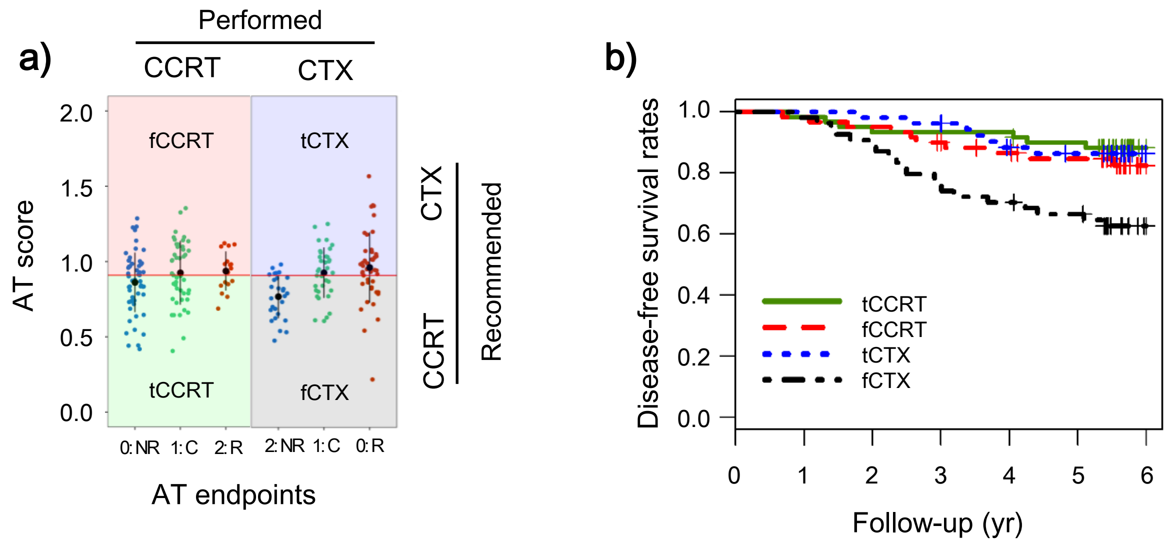

Supplementary Figure S5. Kaplan-Meier plots of ND panel in SEPROGADIC-AT selection module. Patients are divided into four subgroups (true CCRT, false CCRT, true CTX and false CTX) based on median values of the module. (a) AT scores at the three endpoints in the CCRT group (n = 120) and CTX group (n = 107). (b) KM plots of four subtypes. True CCRT (n = 61; observed: 7; green line), false CCRT (n = 59; observed: 10; red line), true CTX (n = 54; observed: 6; blue line), false CTX (n = 53; observed: 21; black line).

a)

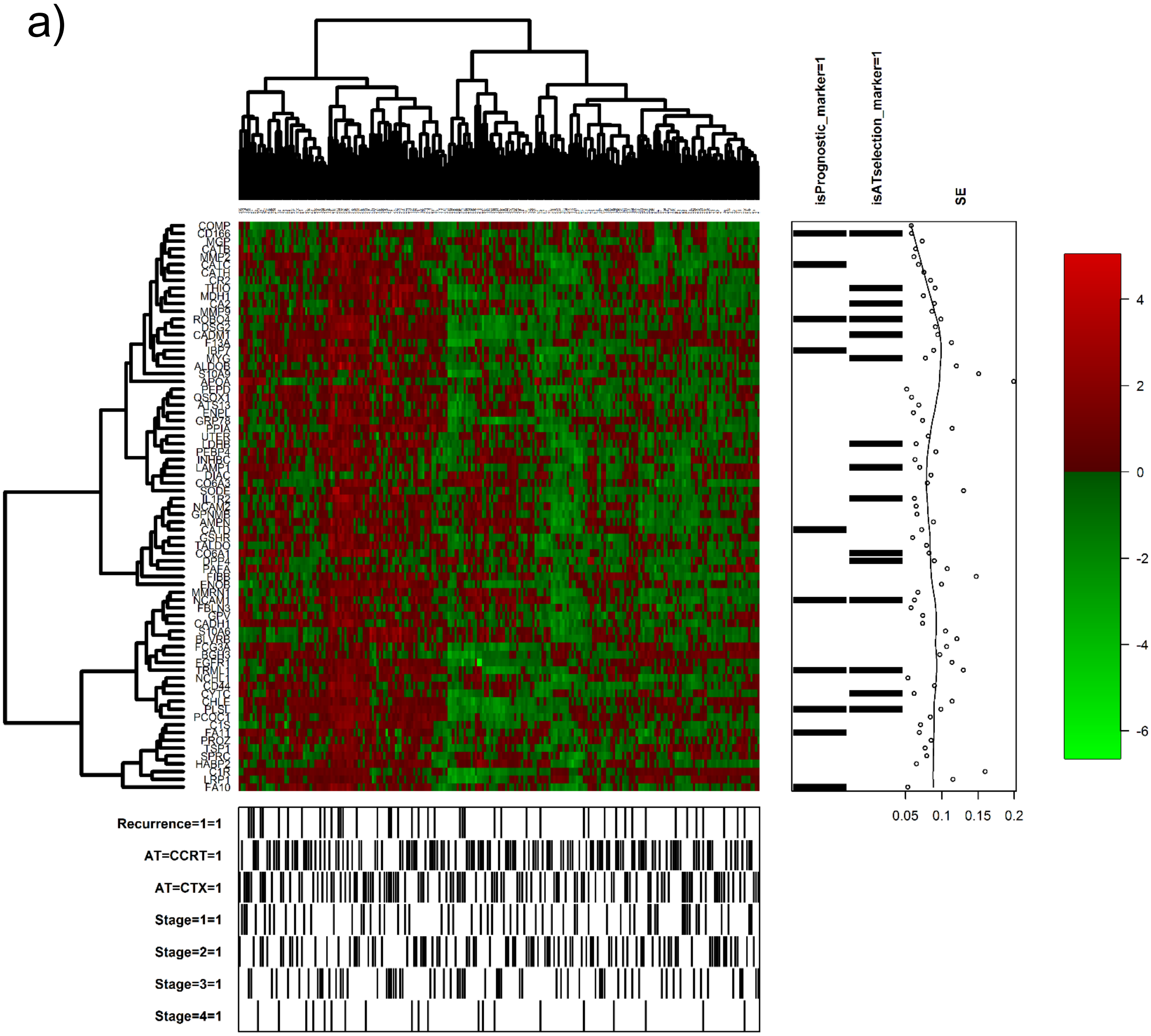

isPrognostic_marker=1
isATselection_marker=1
SE
0.05
0.1
0.15
0.2
4
2
0
-2
-4
-6
Recurrence=1=1
AT=CCRT=1
AT=CTX=1
Stage=1=1
Stage=2=1
Stage=3=1
Stage=4=1

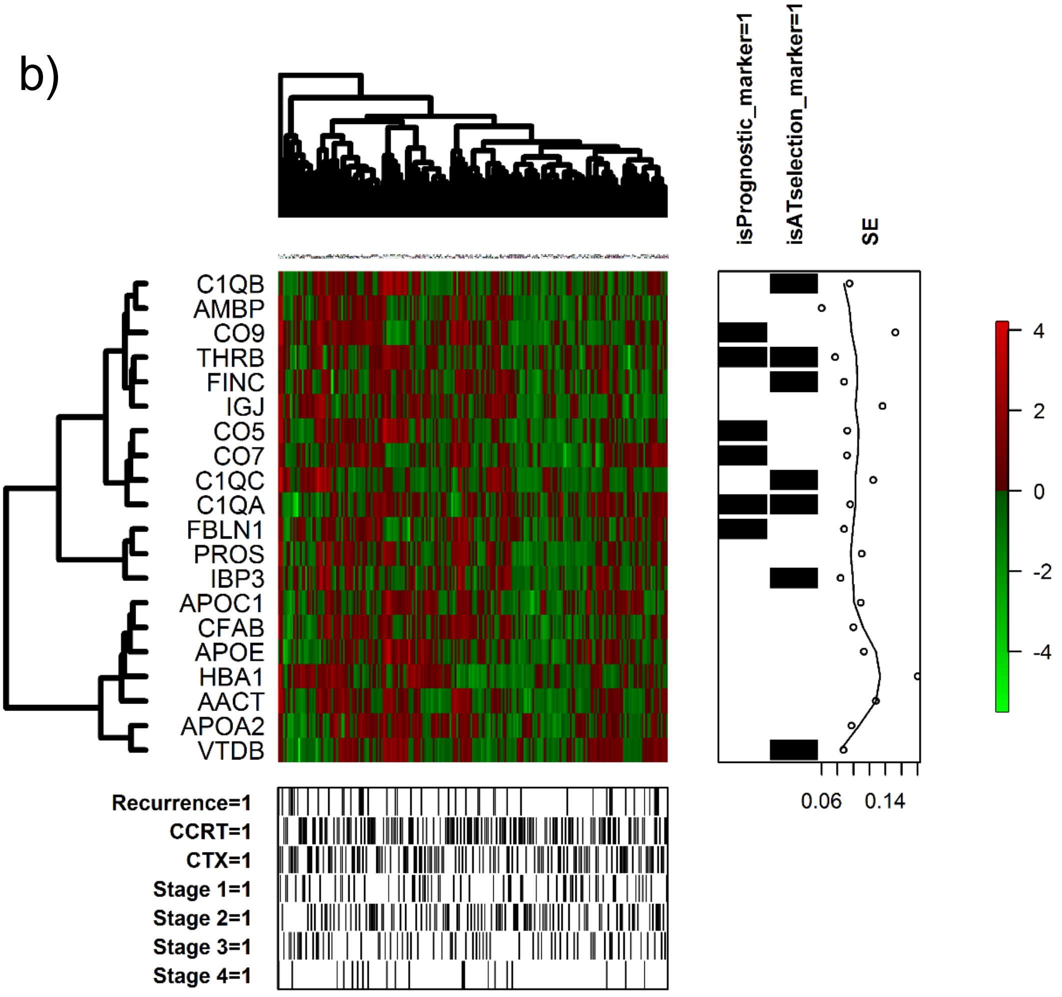

Supplementary Figure S6. Annotated heatmaps with row- and column- dendrograms and annotation for (a) 73 proteins and 227 samples (MD panel) and (b) 20 proteins and 227 samples (ND panel).

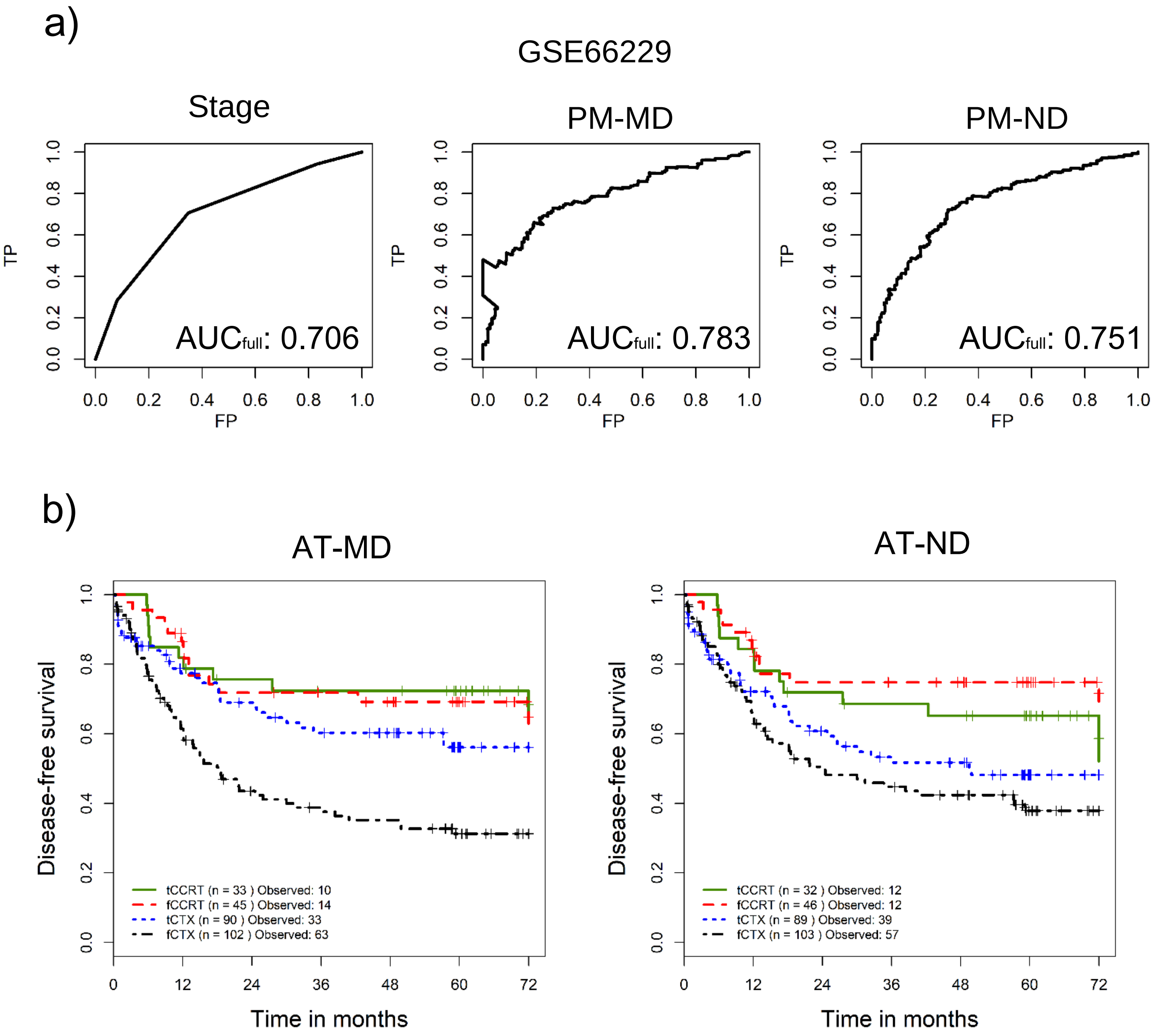

Supplementary Figure S7. Validation of SEPROGADIC modules in the public GEO dataset (GSE 66229). **(a)** SurvivalROC analysis for 270 patients in stage-only model (Stage), SEPROGADIC-PM modules based on MD (PM-MD) and ND (PM-ND) panels. **(b)** K-M plots of SEPROGADIC-AT modules in MD and ND panel.
